# Quantitative modeling of transcription and translation of an all-*E. coli* cell-free system

**DOI:** 10.1038/s41598-019-48468-8

**Published:** 2019-08-19

**Authors:** Ryan Marshall, Vincent Noireaux

**Affiliations:** School of Physics and Astronomy, University of Minnesota, 115 Union Street SE, Minneapolis, MN 55455 USA

**Keywords:** Synthetic biology, Dynamical systems

## Abstract

Cell-free transcription-translation (TXTL) is expanding as a polyvalent experimental platform to engineer biological systems outside living organisms. As the number of TXTL applications and users is rapidly growing, some aspects of this technology could be better characterized to provide a broader description of its basic working mechanisms. In particular, developing simple quantitative biophysical models that grasp the different regimes of *in vitro* gene expression, using relevant kinetic constants and concentrations of molecular components, remains insufficiently examined. In this work, we present an ODE (Ordinary Differential Equation)-based model of the expression of a reporter gene in an all *E. coli* TXTL that we apply to a set of regulatory elements spanning several orders of magnitude in strengths, far beyond the T7 standard system used in most of the TXTL platforms. Several key biochemical constants are experimentally determined through fluorescence assays. The robustness of the model is tested against the experimental parameters, and limitations of TXTL resources are described. We establish quantitative references between the performance of *E. coli* and synthetic promoters and ribosome binding sites. The model and the data should be useful for the TXTL community interested either in gene network engineering or in biomanufacturing beyond the conventional platforms relying on phage transcription.

## Introduction

Cell-free transcription-translation (TXTL) is emerging as a versatile technology to develop, engineer and interrogate biochemical systems programmed with DNA^1^. TXTL is used from the molecular to the cellular scales, in reaction volumes spanning seventeen orders of magnitude, to process DNA programs that are getting larger and larger^2,3^. While an increasing number of laboratories are using this technology to prototype biomolecular systems *in vitro*, simple coarse grained descriptions that capture, in a single set of equations, its basic mechanisms, regimes, and limitations are still missing, although phenomenological observations such as saturation of the TXTL components have been reported^4–7^. The lack of such elementary biophysical models that take into account the concentration of TXTL resources and that deliver measured biochemical constants limits the development of true quantitative work in TXTL, circuit engineering in particular. With the increasing complexity of gene circuits executed *in vitro*, it is essential to define the working principles of TXTL, such as the linear and saturation response regimes of gene expression with respect to the concentration of plasmid, the strengths of the regulatory parts, and the concentration of TX and TL molecular machineries. Such a model can provide the necessary basic quantitative information to better exploit the strengths and advantages of TXTL, and thus execute DNA programs in optimum conditions. The rapid development of TXTL platforms from bacteria other than *E. coli*^8^ also support the need for building up accurate models of *in vitro* DNA-dependent protein synthesis.

Several non-stochastic, quantitative coarse-grained models of hybrid TXTL have been reported^9–12^. For instance, the dynamics of protein synthesis in the PURE system, one of the major TXTL platforms used in the field, is described by a sophisticated model composed of hundreds of biochemical reactions^11,13^. Cell-free protein synthesis in extract-based systems has been recently described, including several metabolic networks for energy regeneration and amino acid biosynthesis^14^. These models provide a description of the conventional T7 hybrid TXTL, where bacteriophage transcription, T7 RNA polymerase and promoter, is coupled to the translation machinery of an organism, *E. coli* for example. The development of versatile TXTL systems with broad transcription repertoires has opened the field to constructing and prototyping DNA programs composed of many regulatory elements with different strengths^5,6,15^, as opposed to the T7 hybrid systems based on just several parts. The synthesis of whole phages, such as T7 and T4^16,17^ demonstrates that an all-*E. coli* TXTL system relying on the endogenous transcription machinery can process remarkably large DNA programs containing tens of regulatory elements with strengths spanning several orders of magnitude. The quantitative description of such TXTL systems has not been sufficiently examined, however, even at the simplest level.

In this work, we present a simple non-stochastic ODE (Ordinary Differential Equation) model of an all-*E. coli* TXTL system^6^, for which we previously described its coarse-grained dynamics^18^. The biophysical model reported in the present article is suitable for cell-free reactions performed in batch mode in volumes on the order of a few microliters. It is the case for a majority of TXTL applications, carried out at the microliter scale or above in well-mixed reactions. This model is applied to a set of three promoters specific to the primary sigma factor 70 (rpoD) in combination with a set of three untranslated regions (UTRs), both spanning a strength of about two orders of magnitude. We determine the rates of protein synthesis in the steady state for the nine combinations with respect to the plasmid concentrations, and to the concentrations of TX and TL molecular components. We test the robustness of the model against several key biochemical constants experimentally determined to constrain the model fitting and simulations. We demonstrate that our model captures the major TXTL regimes and saturations, which are predominantly due to the depletion of ribosomes on the messenger RNAs. Finally, we compare the synthetic sets of promoters and UTRs to a set of natural regulatory parts from *E. coli* so as to establish a reference table of the performances of regulatory elements between TXTL and *in vivo*. In addition to being accessible, the model should facilitate tuning, setting and choosing the strengths and stoichiometry of regulatory parts making circuits.

## Results and Discussion

### Phenomenology

The transcription of the all-*E. coli* TXTL toolbox relies on the core RNA polymerase and the primary sigma factor 70 (RpoD), as discussed previously in several articles^6,19^. All the circuits executed in this system, commercialized under the name myTXTL, are booted up through this transcription mechanism. In our reference plasmid P70a-deGFP, the gene *degfp* encoding the reporter protein deGFP is cloned under the promoter P70a, specific to sigma 70 (Fig. S1). P70a, derived from the phage lambda, is one of the strongest *E. coli* promoters reported so far. The untranslated region (UTR), located between the promoter and the ATG, is the UTR downstream of promoter 14 from the phage T7^20^. It is the strongest bacterial UTR reported so far, and used in many standard plasmids to overexpress proteins in *E. coli*. It is defined as UTR1 in this work. The synthetic transcription terminator T500 is cloned downstream of the *degfp* gene. P70a-deGFP is designated as our reference plasmid because it delivers the strongest gene expression *in vitro*. We compare the performance of single regulatory elements (promoters, UTR, terminators) and of other plasmids to P70a-deGFP.

**Figure 1:**
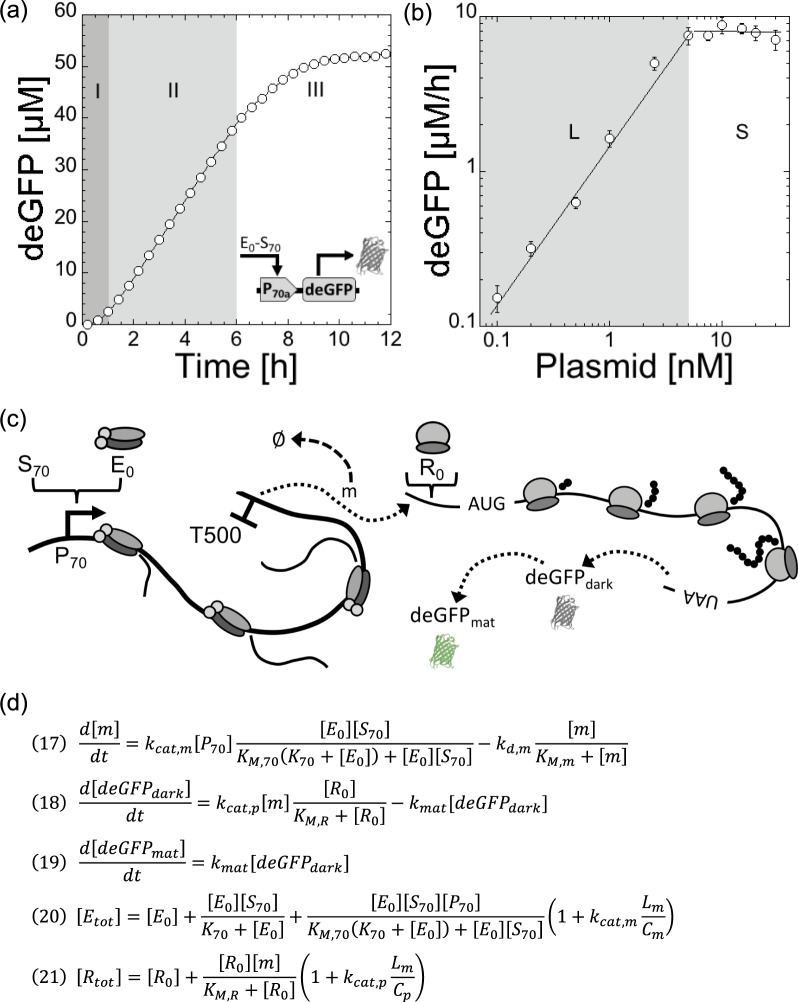
Cell-free expression of the reporter protein deGFP in the all-*E. coli* TXTL system using the plasmid P70a-deGFP. (**a**) Kinetics of deGFP synthesis at 5 nM plasmid showing three regimes: (I) transient regime, (II) steady state, (III) plateau (gene expression stops). (**b**) Maximum rate of deGFP synthesis as a function of the plasmid concentration. Two regimes are observed: linear (L) at low plasmid concentration, saturated (S) at high plasmid concentration. (**c**) Schematic of the model showing most of the components included in the model. (**d**) Final equation set of the model. Equations 20 and 21 have to be solved for E_0_ and R_0_ respectively.

The typical kinetics of deGFP synthesis in a TXTL reaction, using P70a-deGFP as template, shows three phases (Fig. 1a). The first regime, that lasts 30 min to 1 h, is a transient regime when gene expression starts. The second regime, between 1–6 h, corresponds to a steady state. The reporter protein deGFP, which does not degrade in our study, accumulates linearly in time because the concentration of *degfp* messenger RNA (mRNA) is constant. The last regime, typically observed after 6 hours of incubation, is when gene expression curves towards a plateau. This regime is complex to interpret because it corresponds to a depletion of the biochemical building blocks (amino acids, ribonucleosides) and to a change of the biochemical conditions (pH drop for example, see^21^). When the concentration of plasmid P70a-deGFP is varied, the maximum rate of deGFP synthesis in steady state is linearly proportional to the plasmid concentration below 5 nM (Fig. 1b). We observe a saturation of the rate above 5 nM of template. The transition from the linear to the saturated regime is sharp. The linear and saturated regimes observed for the rate of deGFP synthesis are also observed for the protein synthesis yield (Fig. S2). We performed the same experiments with the plasmid P70a-mCherry and observed the same trends for a different reporter protein (Fig. S2). It is this phenomenological observation that we model in this article. We hypothesize that this saturation occurs when either the transcription machinery (core RNA polymerase) or the translation machinery as suggested before^7^, or both, are entirely depleted. For instance, at a sufficiently large concentration of synthesized mRNA, all the ribosomes are performing translation. Therefore, adding more DNA template to the reaction does not convert to more protein produced. As we shall see, transcription in this system never saturates. Our goal is to (i) derive a simple model that captures this hypothesis, (ii) constrain the model by determining experimentally some of the kinetics constants and concentrations, (iii) and test the sensitivity of the model with respect to biochemical parameters.

### Model

The schematic of TXTL of a reporter gene under a constitutive promoter (P70a-deGFP) (Fig. 1c), shows most of the major biochemical species that we include in the model:

- E_0_: free core RNA polymerase
- S_70_: sigma factor 70
- P_70_: promoter specific to sigma 70 (S70)
- m: *degfp* mRNA
- Rnase: ribonucleases responsible for mRNA degradation
- R_0_: free ribosomes
- deGFP_dark_: non-mature deGFP (not fluorescent)
- deGFP_mat_: mature deGFP (fluorescent)
- L_m_: length in nt of the mRNA (or gene)
- C_m_: transcription rate in bp/s
- C_p_: translation rate in b/s

**Figure 2:**
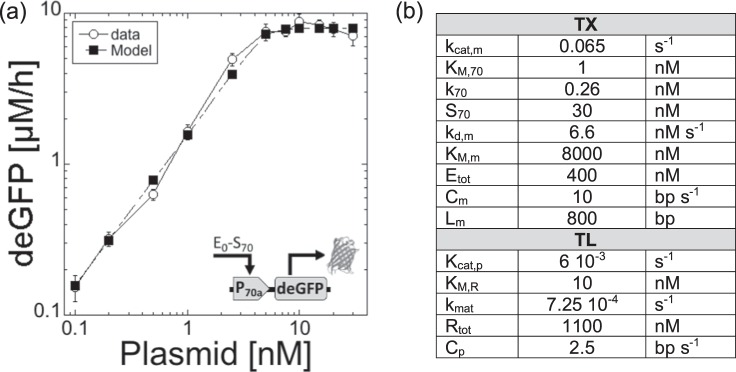
Maximum rate of deGFP synthesis in the all-*E. coli* TXTL system as a function of plasmid concentration (P70a-deGFP). (**a**) Data versus model. (**b**) Biochemical constants for the best fit, shown in (**a**).

The model is based on only three ordinary differential equations (ODEs) and two equations for conservation: the total concentrations of RNA polymerases and ribosomes are constant (Fig. 1d). The biochemical constants and concentrations for our best fit are summarized in the Table Fig. 2. The model is derived using the following appropriate assumptions:

- quasi-steady state for Michaelis-Menten terms. K_M,70_, K_M,m_, and K_M,R_ are the Michaelis-Menten constants for transcription, mRNA degradation and translation respectively.
- nutrients necessary for gene expression (tRNA, amino acids, ribonucleosides) are in infinite supply during the steady state.
- the concentration of holoenzyme RNA polymerase-Sigma 70 is larger than the concentration of template (i.e. larger than the concentration of promoter P70).
- Sigma 70 is not limiting for transcription, which is confirmed by the sensitivity assay.
- the concentration of ribonucleases is smaller than the concentration of synthesized mRNA (m).
- the concentration of ribosomes (R_0_) is larger than the concentration of synthesized mRNA (m).
- translation initiation factors are never limiting.
- the maturation of deGFP_dark_ to deGFP_mat_ is modeled by a first order kinetics, which fits very well to the data in the maturation assay (Supplementary Information).
- none of the components of TX and TL are degraded until the end of the steady state: their concentration is constant. This hypothesis is supported by the fact that this system can be used in semi-continuous mode to express proteins for about a day^6,19^. It is the major difference with respect to the work by Stogbauer and coworkers^10^, whose model attributes saturation of the synthesis rate to a degradation of the TX and TL components.

Using these assumptions, the set of three ODEs that describes the kinetics of deGFP synthesis is the following:

1 $$\frac{d[m]}{dt}={k}_{cat,m}[{P}_{70}]\frac{[{E}_{70}]}{{K}_{M,70}+[{E}_{70}]}-k[{R}_{nase}]\frac{[m]}{{K}_{M,m}+[m]}$$

2 $$\frac{d[deGF{P}_{dark}]}{dt}={k}_{cat,p}[m]\frac{[{R}_{0}]}{{K}_{M,R}+[{R}_{0}]}-{k}_{mat}[deGF{P}_{dark}]$$

3 $$\frac{d[deGF{P}_{mat}]}{dt}={k}_{mat}[deGF{P}_{dark}]$$

The term of mRNA degradation is re-written by taking k [R_nase_] = k_d,m_ (Eq. 4). Based on our previous work^6,22^, mRNA degradation in our system behaves as a first order kinetics which means that K_M,m_ ≫ [m]. The mRNA degradation term is not written as a first order kinetics, however, for modeling purposes (to avoid a negative mRNA concentration in the execution of the Matlab program). The constants k_d,m_ (6.6 nM s^−1^) and K_M,m_ (8000 nM) were chosen so as to obtain k_deg,m_ determined by the assay later described and so that K_M,m_ ≫ [m], which is the case because [m] at the transition from the linear to saturated regimes (5 nM P70a-deGFP) is on the order of 100 nM (Fig. S3). The model is independent from the numerical values of k_d,m_ and K_M,m_ as long as their ratio is equal to k_deg,m_ and K_M,m_ ≫ [m].

4 $$\begin{array}{l}k[{R}_{nase}]\frac{[m]}{{K}_{M,m}+[m]}={k}_{d,m}\frac{[m]}{{K}_{M,m}+[m]}\\ (\approx {k}_{deg,m}[m]\,with\,{k}_{deg,m}\approx \frac{{k}_{d,m}}{{K}_{M,m}}\,and\,{K}_{M,m}\gg [m])\end{array}$$

The set of Equations (1–3) becomes:

5 $$\frac{d[m]}{dt}={k}_{cat,m}[{P}_{70}]\frac{[{E}_{70}]}{{K}_{M,70}+[{E}_{70}]}-{k}_{d,m}\frac{[m]}{{K}_{M,m}+[m]}$$

6 $$\frac{d[deGF{P}_{dark}]}{dt}={k}_{cat,p}[m]\frac{[{R}_{0}]}{{K}_{M,R}+[{R}_{0}]}-{k}_{mat}[deGF{P}_{dark}]$$

7 $$\frac{d[deGF{P}_{mat}]}{dt}={k}_{mat}[deGF{P}_{dark}]$$

In the next step we build two equations of conservation for the core RNA polymerases and ribosomes. The sigma factor 70 has two forms, free (S_70free_) or complexed with the core RNA polymerase (E_70_):

8 $$[{S}_{70}]=[{S}_{70free}]+[{E}_{70}]$$

We consider that the following biochemical reaction is at equilibrium all the time (i.e. it is a fast biochemical reaction with respect to the others): We call K_70_ the dissociation constant:

9 $${S}_{70free}+{E}_{0}\mathop{\leftrightarrow }\limits_{{K}_{70}}{E}_{70}$$

Therefore, using Eq. (8):

10 $$[{E}_{70}]=\frac{[{E}_{0}][{S}_{70free}]}{{K}_{70}}=\frac{[{E}_{0}][{S}_{70}]}{{K}_{70}+[{E}_{0}]}$$

The core RNA polymerase has three forms: free (E_0_), complexed with S_70_ (E_70_), or performing transcription on mRNA (E_m_). E_tot_ is constant:

11 $$[{E}_{tot}]=[{E}_{0}]+[{E}_{70}]+[{E}_{m}]$$

The number of core RNA polymerases that are bound to DNA is (see)^23^:

12 $$[{E}_{m}]=\frac{[{E}_{70}][{P}_{70}]}{{K}_{M,70}+[{E}_{70}]}(1+{k}_{cat,m}\frac{{L}_{m}}{{C}_{m}})=\frac{[{E}_{0}][{S}_{70}][{P}_{70}]}{{K}_{M,70}({K}_{70}+[{E}_{0}])+[{E}_{0}][{S}_{70}]}(1+{k}_{cat,m}\frac{{L}_{m}}{{C}_{m}})$$

The first term in Eq. 12 corresponds to the core RNA polymerase on the promoter and the other term the core RNA polymerases that have engaged in transcription. We then get the conservation equation, Eq. 13, that has to be solved for E_0_:

13 $$[{E}_{tot}]=[{E}_{0}]+\frac{[{E}_{0}][{S}_{70}]}{{K}_{70}+[{E}_{0}]}+\frac{[{E}_{0}][{S}_{70}][{P}_{70}]}{{K}_{M,70}({K}_{70}+[{E}_{0}])+[{E}_{0}][{S}_{70}]}(1+{k}_{cat,m}\frac{{L}_{m}}{{C}_{m}})$$

We proceed in a similar manner to construct the conservation of ribosomes. Note that here we assume that the translation initiation and termination factors are not limiting the process of translation. Ribosomes can be in two forms, free (R_0_), and performing translation on mRNA (R_m_):

14 $$[{R}_{tot}]=[{R}_{0}]+[{R}_{m}]$$

The number of ribosomes on mRNA is:

15 $$[{R}_{m}]=\frac{[{R}_{0}][m]}{{K}_{M,R}+[{R}_{0}]}(1+{k}_{cat,p}\frac{{L}_{m}}{{C}_{p}})$$

The first term in Eq. 15 corresponds to the ribosomes on the ribosome binding site and the other term is for the ribosomes that have engaged into translation. Eq. 16 that has to be solved for R_0_:

16 $$[{R}_{tot}]=[{R}_{0}]+\frac{[{R}_{0}][m]}{{K}_{M,R}+[{R}_{0}]}(1+{k}_{cat,p}\frac{{L}_{m}}{{C}_{p}})$$

The final system of equations (using Eqs (5–7) and 10) is (also shown in Fig. 1d):

17 $$\frac{d[m]}{dt}={k}_{cat,m}[{P}_{70}]\frac{[{E}_{0}][{S}_{70}]}{{K}_{M,70}({K}_{70}+[{E}_{0}])+[{E}_{0}][{S}_{70}]}-{k}_{d,m}\frac{[m]}{{K}_{M,m}+[m]}$$

18 $$\frac{d[deGF{P}_{dark}]}{dt}={k}_{cat,p}[m]\frac{[{R}_{0}]}{{K}_{M,R}+[{R}_{0}]}-{k}_{mat}[deGF{P}_{dark}]$$

19 $$\frac{d[deGF{P}_{mat}]}{dt}={k}_{mat}[deGF{P}_{dark}]$$

20 $$[{E}_{tot}]=[{E}_{0}]+\frac{[{E}_{0}][{S}_{70}]}{{K}_{70}+[{E}_{0}]}+\frac{[{E}_{0}][{S}_{70}][{P}_{70}]}{{K}_{M,70}({K}_{70}+[{E}_{0}])+[{E}_{0}][{S}_{70}]}(1+{k}_{cat,m}\frac{{L}_{m}}{{C}_{m}})$$

21 $$[{R}_{tot}]=[{R}_{0}]+\frac{[{R}_{0}][m]}{{K}_{M,R}+[{R}_{0}]}(1+{k}_{cat,p}\frac{{L}_{m}}{{C}_{p}})$$

We did not include protein degradation in the experiments. There are two reasons for this. First, protein degradation, achieved by the ClpXP complex in TXTL, is a zeroth order kinetic reaction that does not allow a steady state for proteins^6^. Consequently, the analysis is less interesting. Second, the concentration of ClpXP complex does not seem to remain constant in the TXTL reaction (data not shown), presumably due to the well-established instability of ClpX^24^. That would make the analysis and modeling complicated and phenomenological.

### TX

The biochemical constants and other parameters (for our best fit) are summarized in the Table Fig. 2b. In its simple expression, the initiation frequency k_TX_ for TX depends on k_cat,m_, K_M,70_ and E_70_ (Eqs 5 and 22). k_TX_ varies over three orders of magnitude^25^, with a maximum that can reach 30 initiations per 60 seconds^26,27^. This puts a limit on k_cat,m_ to 0.5 s^−1^, especially at low plasmid concentration when free RNA polymerase (E_0_) is an infinite reservoir and E_70_ equals S_70_. The rate constant for mRNA synthesis k_cat,m_ was estimated to be between 10^−1^ and 10^−3^ s^−1^ for *E. coli* promoters^25^. For a strong promoter like P70a, we expect k_cat,m_ to be at the high end of these estimations. In our best fit, k_cat,m_ = 0.065 s^−1^. The Michaelis-Menten constant K_M,70_ is typically between 1 nM and 100 nM^25,28^. In our previous TXTL work^22^, based on the first version of the system^5^, K_M,70_ was estimated to be around 10 nM for the promoter P70a. In this work, we used the new version of this TXTL system^6^; our best fit was with K_M,70_ = 1 nM. The concentration of core RNA polymerases in *E. coli* varies between 1500 and 11400 molecules per cell depending on the growth conditions^26^. Because the lysate is prepared from cells growing in a rich medium and collected in the exponential phase, the concentration of core RNA polymerase in the collected cells is considered to be on the high end at about 11000–12000 per cell. Taking into account a dilution factor of about 7–10 during the lysate preparation (200–320 mg/ml of proteins in the *E. coli* cytoplasm^29^, 30 mg/ml for the lysate), the maximal concentration of core RNA polymerase is around 1.5 µM if all the enzymes are released during the preparation. This estimation translates as a maximum of E_tot_ = 500 nM of core RNA polymerase in a TXTL reaction, which contains a 1/3 volume fraction of lysate. The minimum concentration of free core RNA polymerase in TXTL is found by only considering the polymerases not bound to DNA^6,30^. Our best fit was found for E_tot_ = 400 nM. The same calculation was made for the primary sigma factor 70 (RpoD), whose number density is around 500–700 copies per cell (about 500–700 nM for a cell volume of 1 femtoliter)^31,32^. In a TXTL reaction, sigma 70 is therefore at a maximum concentration of about S_70_ = 30–35 nM, which works for our best fit. The dissociation constant between sigma 70 and the core RNA polymerase has been precisely determined: K_70_ = 0.26 nM^32^. The rate constant of the deGFP mRNA degradation was determined by an assay (Fig. S4): 1/k_deg,m_ = 8.25 10^−4^ s (20.2 min for the mean lifetime). This constant was written as k_deg,m_ = k_d,m_/K_M,m_ (Eq. 4) with k_d,m_ = 6.6 nM/s and K_M,m_ = 8000 nM. The concentration of promoter P_70_ and gene (both equal to the plasmid concentration) was fixed experimentally. The length of the transcribed gene is L_m_ = 750 bp, from the TX start to the TX terminator. The average speed of TX (speed of the core RNA polymerase on DNA) in the all *E. coli* TXTL was estimated by an assay (Fig. S5): C_m_ ≈ 10 bp/s, which is about 4–8 times smaller than *in vivo*^26^. E_0_, the concentration of free core RNA polymerase, is determined by Eq. 20.

The mRNA steady state [m]_SS_ (Eq. 23) is found by setting Eq. 17 to zero (Eq. 22). For low plasmid concentration (in the linear regime), one can assume that E_70_ ≫ K_M,70_ (or that K_70_ ≪ E_0_) and therefore k_cat,m_ ≈ k_TX_. The mRNA mean lifetime 1/k_deg,m_ for the malachite green aptamer (MGapt) was estimated using an assay (Fig. S6): 1/k_deg,m_ ≈ 27 min. Our measurements of [m]_SS_ at low plasmid concentration, using the malachite green aptamer as an RNA probe (Fig. S7), gives us a value of k_cat,m_ ≈ k_TX_ = 1.5 10^−2^ s^−1^ using [m]_SS_ = 25 nM at 1 nM plasmid. This experiment, however, can only provide a low estimation for this constant (i.e. the value for k_TX_ can only be underestimated because the assay may not report all the malachite green aptamers synthesized or fluorescent). In our simulations, we found that the best fit was obtained with k_cat,m_ ≈ k_TX_ = 6.5 10^−2^ s^−1^ (Fig. 2).

22 $$\begin{array}{rcl}{\frac{d[m]}{dt}} & = & {{k}_{TX}[{P}_{70}]-{k}_{deg,m}[m]}\\&  & {with:{k}_{TX}\,=\,{k}_{cat,m}\frac{[{E}_{70}]}{{K}_{M,70}+[{E}_{70}]}}\,\,\,\,{=}\,\,\,\,{{k}_{cat,m}\frac{[{E}_{0}][{S}_{70}]}{{K}_{M,70}({K}_{70}+[{E}_{0}])+[{E}_{0}][{S}_{70}]}}\\&  & {and:{k}_{deg,m}\,=\,\frac{{k}_{d,m}}{{K}_{M,m}}}\end{array}$$

23 $${[m]}_{SS}=\frac{{k}_{TX}}{{k}_{deg,m}}[{P}_{70}]\approx \frac{{k}_{cat,m}}{{k}_{deg,m}}[{P}_{70}]$$

Note that for the deGFP mRNA, 1/k_deg,m_ ≈ 20 min (Fig. S4), using k_cat,m_ ≈ k_TX_ = 6.5 10^−2^ s^−1^, we get that [m]_SS_ ≈ 80 nM at 1 nM plasmid. A maximum theoretical value (1 nM plasmid ≈ 1 copy per *E. coli*) of [m]_SS_ ≈ 600 nM in TXTL is obtained by taking k_cat,m_ ≈ k_TX_ = 0.5 s^−1^ and a 1/k_deg,m_ ≈ 20 min. Experimentally, one can see that the TX machinery is never limiting in the system because the rate of mRNA synthesis keeps increasing even at plasmid (P70a-deGFP-MGapt) concentrations larger than 5 nM (Fig. S8). As we shall see below, it is the TL machinery that is limiting in the system, i.e. ribosomes are entirely depleted onto the mRNA at plasmid concentrations above 5 nM (P70a-deGFP). Because it is the strongest promoter-UTR pair, the protein synthesis rate or yield for any other promoter-UTR regulatory element is linear with respect to plasmid concentration up to 5 nM or more; saturation of the protein synthesis rate cannot be observed below 5 nM plasmid.

### TL

Similarly to TX, in its simple expression, the initiation frequency k_TL_ (Eq. 24) for TL depends on both k_cat,p_ and K_M,R_, and R_0_. The translation initiation frequency can be as high as 0.5 s^−1^ ^33^. The Michaelis-Menten constant for translation was measured *in vitro* and estimated to be around 23 nM for the 70S ribosome with no tRNA and 10 nM with tRNA^34^. In a previous cell-free system, K_M,R_ was fitted at 65.8 nM^10^. K_M,R_ = 10 nM was used for our best fit. No estimation of the rate constant for protein synthesis k_cat,p_ was found in the literature. At low mRNA concentration, one can expect that R_0_» K_M,R_, which puts a limit on k_cat,p_ to 0.5 s^−1^. The rate constant for the maturation of deGFP was determined by an assay described previously^6^ and repeated in this work (Fig. S9). The average concentration of ribosomes in *E. coli* cells growing in a rich medium, with a doubling time between 20 and 30 minutes, is between 44000 and 73000^26^, which corresponds to 1450–2500 nM in a TXTL reaction. It is in excellent agreement with respect to previous measurements in cell-free systems^35^. R_tot_ = 1100 nM was our best fit for active ribosomes in TXTL. Finally, we estimated the average translation speed (speed of ribosomes on mRNA) to be at least 1 amino acid s^−1^ (2.5 bp s^−1^) (Fig. S10).

24 $$\frac{d[deGF{P}_{dark}]}{dt}={k}_{TL}[m]-{k}_{mat}[deGF{P}_{dark}]\,with\,{k}_{TL}={k}_{cat,p}\frac{[{R}_{0}]}{{K}_{M,R}+[{R}_{0}]}$$

The steady state for deGFP_dark_ is:

25 $${[deGF{P}_{dark}]}_{SS}=\frac{{k}_{cat,p}}{{k}_{mat}}{[m]}_{SS}\frac{1}{1+{K}_{M,R}/[{R}_{0}]}$$

For low plasmid concentrations [P_70_] < 1 nM, one can expect that K_M,R_/R_0_ ≪ 1, therefore:

26 $${[deGF{P}_{dark}]}_{SS}=\frac{{k}_{cat,p}}{{k}_{mat}}{[m]}_{SS}\approx \frac{{k}_{cat,p}}{{k}_{mat}}\frac{{k}_{cat,m}}{{k}_{deg,m}}[{P}_{70}]\,(\mathrm{for}\,[{P}_{70}] < 1\,{\rm{nM}})$$

A simple expression for the linear accumulation of deGFP_mat_ at low plasmid concentration is then:

27 $$\,[deGF{P}_{mat}]\approx \frac{{k}_{cat,p}\,{k}_{cat,m}}{{k}_{deg,m}}[{P}_{70}]\times ({\rm{t}})$$

At 1 nM plasmid P70a-deGFP, we measure a maximum protein synthesis rate of 0.5 nM/s, which indicates that the product k_cat,p_*k_cat,m_ = 4 10^−4^ s^−2^ (taking k_deg,m_ = 8.25 10^−4^ s^−1^ for the deGFP mRNA). The value for k_cat,p_ = 6 10^−3^ s^−1^ was chosen based on this calculation using k_cat,m_ = 6.5 10^−2^ s^−1^. A maximum theoretical value (1 nM plasmid ≈ 1 copy per *E. coli*) of 300 nM/s for the protein synthesis rate in TXTL is obtained by taking k_cat,m_ ≈ k_cat,p_ = 0.5 s^−1^ and a 1/k_deg,m_ ≈ 20 min. As shown for plasmid P70a-deGFP concentrations of 1, 5 and 10 nM, the model also delivers reliable kinetics at steady state for the first few hours, below and above the transition from linear to saturated regimes (Fig. S11). A major hallmark of our approach is how the model grasps very well the sharpness between the linear and saturated regime (Fig. 2). A model describing a similar TXTL system, yet based on a different regeneration system, attributes the saturation to metabolic processes and energy efficiency^14^. When applied to P70a-deGFP, however, this approach neither captures the linear regime nor the sharpness of the response that we observed in this work (see Fig. S1 in^14^). We assume that the behavior of cell-free expression (e.g. presence of a linear response regime and sharpness of the transition from linear to saturated) in both systems do not have the same origin.

### Parts combinations and sensitivity analysis

**Figure 3:**
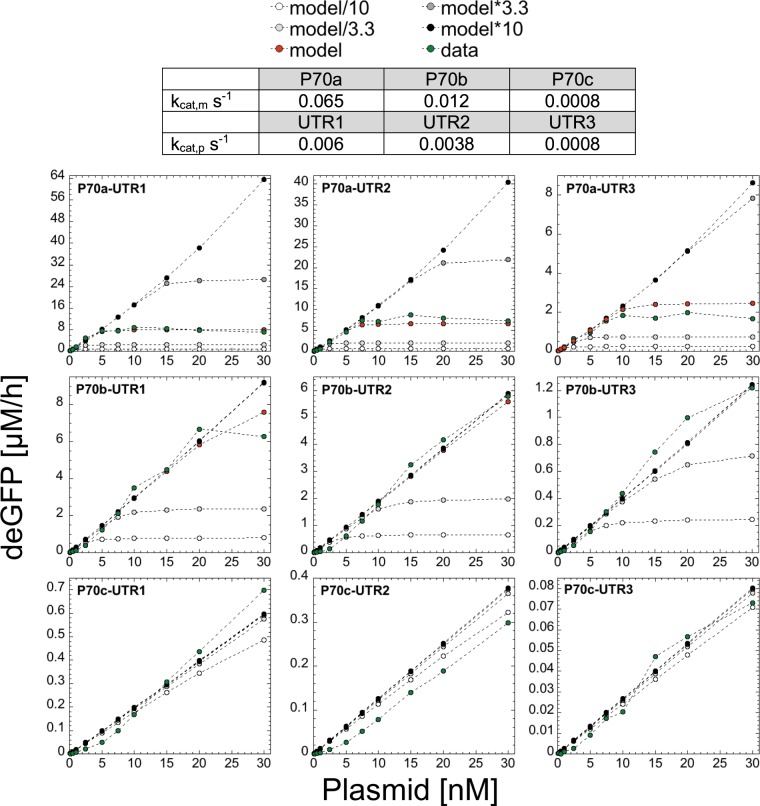
Model Sensitivity to changing the ribosome concentration (1100 nM ribosome as best fit numerical value). Model results for combinations of three promoters and UTRs for the four different concentrations of total ribosomes (/10, /3.33, *3.33, *10) in addition to the best fit. Model*10 means that the concentration of ribosomes is 10*1100 = 11000 nM. Note that when the red dots (model) are not visible, it means that they overlap with the black dots (model*10).

We designed two other promoters, P70b and P70c, derived from P70a (strengths: P70a > P70b > P70c) and two other untranslated regions, UTR2 and UTR3, derived from UTR1 (strengths: UTR1 > UTR2 > UTR3) to create a set of nine combinations (sequences in Supplementary Information). The −35 and −10 of P70a were mutated to get P70b and P70c. The ribosome binding site in UTR1 was mutated to get UTR2 and UTR3. These sets span two orders of magnitude in strengths. By changing the promoter and UTR strengths, we change the value of k_cat,m_ and k_cat,p_, and of K_M,70_ and K_M,R_. Many k_cat,m_-K_M,70_ and k_cat,p_-K_M,R_ pairs can be found to fit the results. However, because the system is only weakly sensitive to changes in the magnitude of the Michaelis-Menten contants K_M,70_ and K_M,R_ (see thereafter), we only changed the value of k_cat,m_ and k_cat,p_ that we determined through the simulations to get the best fits (Fig. 3). We experimentally determined the rate of protein synthesis for the nine combinations with respect to plasmid concentration and performed sensitivity analysis on six biochemical parameters. The sensitivity analysis comprised of varying each of the six biochemical constants, while keeping all the others constants at their best numerical fit values, by one order of magnitude above and below the best fit value. As discussed for P70a-UTR1, translation is the limiting process responsible for saturation of the protein synthesis rate as plasmid concentration is increased. Consequently, the model and data are most sensitive to the ribosome concentration, especially for strong promoters (Fig. 3). As expected, for weak promoters and/or UTRs (e.g. P70c), the response is linear for any plasmid concentration (up to 30 nM tested in this work). In addition to the ribosome concentration, high sensitivity is observed for k_deg,m_ (Fig. S12). As expected, if k_deg,m_ is larger, the system does not saturate and the response remains linear. Conversely, if k_deg,m_ is smaller, the systems saturates more quickly with respect to plasmid concentration. Some sensitivity is observed for k_mat_ (Fig. S13) and for E_tot_ (Fig. S14). Note that for E_tot_, saturation is not observed in the experiments (Fig. S8) as captured by the model. Limitations due to E_tot_ in the plasmid range 0–30 nM (P70a-deGFP) would be observed if E_0_ < 100 nM. The model shows very weak sensitivity to K_M,70_ and K_M,R_ (Figs S15 and S16). The model was not sensitive to changes in S_70_ (Fig. S17). For P70a-deGFP, the model predicts a sharp transition in the concentration of free ribosomes around 5 nM plasmid, while the concentration of free core RNA polymerase decreases sharply only at plasmid concentrations of about 50 nM (Fig. S18).

### Strengths of synthetic vs natural regulatory elements

**Figure 4:**
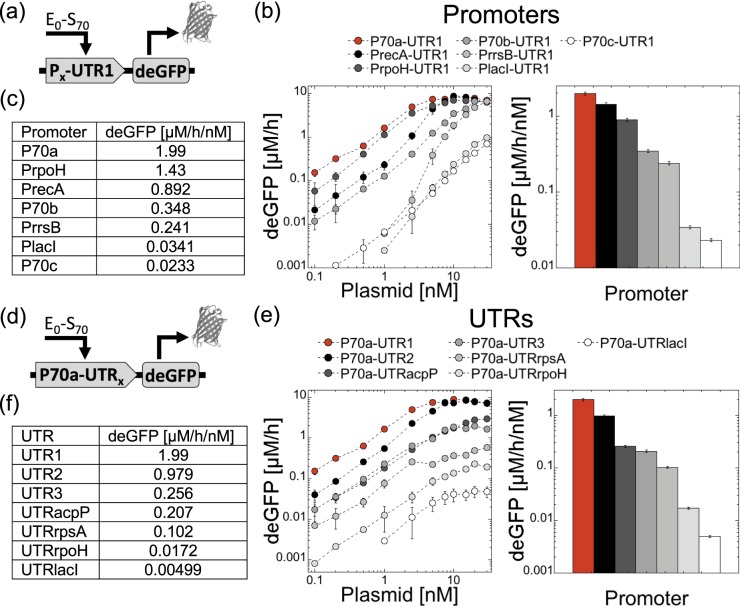
Rates of deGFP synthesis for a synthetic and natural sets of promoters and UTRs. (**a**) Plasmid construction for promoters. (**b**) Rates and maximum rates per plasmid concentration for promoters. (**c**) Table summarizing the values of maximum rates per plasmid concentration for promoters. (**d**) Plasmid construction for UTRs. (**e**) Rates and maximum rates per plasmid concentration for UTRs. (**f**) Table summarizing the values of maximum rates per plasmid concentration for UTRs.

Our next step consisted of testing natural promoters and UTRs from *E. coli* to establish quantitative references with respect to the synthetic parts used to develop the model. Note that the strengths of some promoters have been already compared *in vivo* and *in vitro*^36^. We chose the constitutive promoters of the following genes, some based on protein abundance^37^, that we isolated by coupling each of them to the strong UTR1 (Fig. 4): lacI, rpoH, rrsB, recA. We chose the UTRs of the following genes that we isolated by coupling each of them to the strong promoter P70a (Fig. 4): lacI, rpoH, rpsA, acpP. We measured the rates of deGFP synthesis for all these constructions over the same plasmid range, from 0 to 30 nM (Fig. 4). Most of these constructions showed a linear regime followed by a saturation. Only PrrsB (16S ribosomal RNA promoter) behaved differently with a response curve characterized by a sigmoidal response at low plasmid concentration. As expected, weak promoters such as PlacI never saturate. As importantly, we defined the rates of deGFP synthesis per plasmid concentration (deGFP/h/nM), for each construction in the linear regime, as an indicator of the promoter or UTR strengths (Fig. 4). Many other promoters and UTRs can be rapidly tested in TXTL using this method. This table serves as a minimal quantitative reference between several synthetic promoters/UTRs used in TXTL and natural ones.

### TXTL load calculator

**Figure 5:**
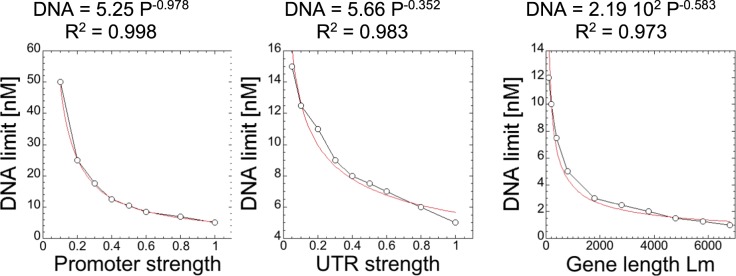
Model predictions for the approximate limiting DNA concentration for different promoter strengths, UTR strengths, and lengths of gene. Each data set was constructed by varying each specific constant (k_cap,m_, k_cat,p_, L_m_) and fit to a power function, then combined to form the final Eq. (28). The factor of 250 in Eq. (28) is due to the length of gene not being normalized, 2.19 10^2^ is divided by the limiting DNA concentration at the length of deGFP (800 nt), which is 4.43 nt. That value is multiplied by the limiting DNA concentration for P70a-deGFP, where P = 1, U = 1, and L_m_ = 800, which is 5 nM. The power function is chosen only because it is a good fit phenomenologically, not for some physiological reasons. The load calculator is just a tool to approximate when the resources will become limiting and the protein synthesis rate will not be in the linear regime. The obtained value tells us how sensitive each parameter is to the limiting DNA concentration, such that they can be combined in one single TXTL load calculator equation.

The last step of this work consisted of building a load calculator as a procedure and formula to determine the burden on the TXTL components, especially on the translation machinery. This approach requires making several plasmids to define the strengths of the parts and measuring the protein synthesis rate (using eGFP for instance) to define the linear and saturated regimes. In order to determine the concentration of DNA (nM) in a TXTL for which the translation machinery will limit the deGFP synthesis rate, we developed an equation that takes into account the promoter strength (P), the UTR strength (U) and the length of the gene being expressed (L_m_) in the DNA construct. The equation was constructed by fitting power function to each variable individually against the approximate concentration of DNA for which the ribosomes became limiting based on the model (Fig. 5). The three fit equations were then combined to form the equation below, which accounts for variations in each of the three variables. In order to make use of the equation, the promoter and UTR strength must already be characterized. P is the strength of the promoter relative to P70a, where P70a is given as a strength of 1. U is the strength of the UTR relative to UTR1, where UTR1 is given as a strength of 1. L_m_ is the length of the gene being expressed in nucleotides. The construction of the equation is detailed further in the Fig. 5.

28 $$[DNA]=250\times {P}^{-0.987}\times {U}^{-0.352}\times {L}_{m}^{-0.583}\approx \frac{250\times {U}^{-0.352}\times {L}_{m}^{-0.583}}{P}$$

If more than one DNA construct is being used in the TXTL reaction and a user wants to know if ribosomes will be limiting, the equation can be used to calculate approximately what fraction of the ribosomes will be used by each DNA construct. For example, if two DNA constructs will be used in a TXTL reaction, and if the equation determines that the limiting concentration of one DNA construct alone is 5 nM, and 1 nM will be used in the reaction, then the limiting concentration of the second DNA construct should be reduced by 1 nM/5 nM = 20%. This process can be repeated if more than two DNA constructs are being used in a TXTL reaction.

## Conclusions

As the field of cell-free expression is rapidly growing, developing models with constrained biochemical parameters is necessary to determine the TXTL biochemical regimes and provide users with quantitative information to set the strengths and stoichiometry of regulatory parts making circuits, either executed in batch mode reactions or other settings such as microfluidics chips and synthetic cells. Because each cell-free system is different, model should be specific and accompanied by relevant measurements for each platform. In this work, our model captures remarkably well the linear and saturated regime, and more importantly, the sharpness of the transition between the two regimes for the all-*E. coli* system. While powerful computer tools are available to develop complex and sophisticated models, some models should also remain practical and thus accessible.

## Materials and Methods

### TXTL reactions

The TXTL system used in this work is the myTXTL kit from Arbor Biosciences. This system has been described in several articles^6,19^. TXTL reactions were assembled using a Labcyte Echo 550 Acoustic Liquid Handler, to volumes of 2 µl, and incubated at 29 °C. At a scale of 2 µl, the reactions were not limited by oxygen consumption. Individual TXTL reaction components were added to the 384 well source plate (Labcyte PP-0200), dispensed into a 96 well v-bottom plate (Sigma-Aldrich CLS-3857) and sealed with a well plate storage mat (Sigma-Aldrich CLS-3080). Protein fluorescence kinetics measurements were performed with the reporter plasmid P70a-deGFP, expressing the truncated version of eGFP (25.4 kDA, 1 mg/mL = 39.38 µM)^19^. deGFP fluorescence was measured on either a Biotek Neo2 or Biotek H1 plate reader at excitation and emission wavelengths of 485 nM and 528 nM, respectively, typically measuring every 3 minutes for 16 hours, with an incubation temperature of 29 °C. Fluorescence on the plate readers was calibrated using pure eGFP (Cell Biolabs STA-201) following a procedure described previously^6^. MG aptamer RNA fluorescence kinetics measurements were performed with 20 µM malachite green dye, and using excitation and emission wavelengths of 620 nM and 660 nM, respectively. Each data set was repeated at least three times. Error bars represent the standard deviations among the repeats.

### DNA constructions

Plasmids were constructed using standard restriction enzyme cloning techniques. The sequences of the DNA constructions used in this work can be found in the Supplementary Information. Plasmids were amplified using DH5alpha chemically competent cells, isolated with a standard plasmid midi prep kit, and spin-column purified with a standard PCR purification kit. The extra purification step ensures that the plasmid is the cleanest possible, as required for TXTL experiments.

### Assays

The Supplementary Information contains the description of the following assays: maturation time of deGFP (based on^6^); deGFP mRNA mean lifetime (based on^6^); transcription speed (C_m_) and translation speed (C_p_); malachite green aptamer degradation rate.

### Matlab codes

An example of Matlab code is given in the Supplementary Information.

## Supplementary information

SI

## Data Availability

The datasets generated during and/or analyzed during the current study are available from the corresponding author on reasonable request.
