# Supplementary material for: Quantitative modeling of transcription and translation of an all-E. coli cell-free system
Source: Sci Rep. 2019 Aug 19;9:11980. doi: 10.1038/s41598-019-48468-8 (PMC6700315; doi:10.1038/s41598-019-48468-8)
Supplement: Supplementary file 1 — SI [file 41598_2019_48468_MOESM1_ESM.pdf]

Supporting Information

Quantitative modeling of transcription and translation of an all-*E. coli* cell-free system

Ryan Marshall and Vincent Noireaux
School of Physics and Astronomy, University of Minnesota, 115 Union Street SE, Minneapolis, MN 55455, USA

Correspondence: noireaux@umn.edu

Content:

Figure S1	P70a-deGFP sequence	2
Figure S2	Rate and total synthesis of deGFP (P70a-deGFP)	3
Figure S3	P70a-deGFP-Mgapt sequence	4
Figure S4	deGFP mRNA mean lifetime	5
Figure S5	Estimation of transcription speed (C_m)	7
Figure S6	MG aptamer mRNA mean lifetime	8
Figure S7	kinetics of MG apt accumulation	9
Figure S8	mRNA synthesis never saturates	10
Figure S9	Estimation of deGFP maturation time	11
Figure S10	Estimation of the translation speed (C_p)	13
Figure S11	Kinetics of deGFP synthesis for three plasmid concentrations	14
Figure S12	Sensitivity to changing the deGFP mRNA degradation rate $k_{deg,m}$	15
Figure S13	Sensitivity to changing the deGFP maturation rate k_{mat}	16
Figure S14	Sensitivity to changing the concentration of core RNA polymerase E_{tot}	17
Figure S15	Sensitivity to changing the Michaelis-Menten constant for transcription $K_{M,70}$	18
Figure S16	Sensitivity to changing the Michaelis-Menten constant for transcription $K_{M,R}$	19
Figure S17	Sensitivity to changing the concentration of sigma factor 70	20
Figure S18	Model prediction concentrations of free ribosomes R_0 and RNA polymerase E_0	21
DNA sequences		22
Matlab code		24
References		26

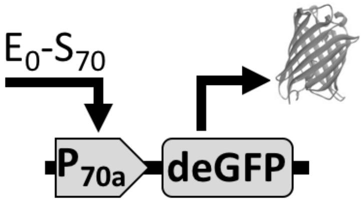

GCATGCTGAGCTAACACCGTGCGTG**TTGACA**ATTTTACCTCTGGCGGT**GATAAT**G
GTTGCaGCTAGC*AATAATTTTGTTTAACTTTAAGAAGGAGATATACC*ATGGAGCTTTTCA
CTGCGTTGTTCCCATCCTGGTCGAGCTGGACGGCGACGTAAACGGCCACAAGTTC
AGCGTGTCCGGCGAGGGCGAGGGCGATGCCACCTACGGCAAGCTGACCCTGAAGT
TCATCTGCACCACCGGCAAGCTGCCCGTGCCCTGGCCCACCCTCGTGACCACCCTG
ACCTACGGCGTGCAGTGCTTCAGCCGCTACCCCGACCACATGAAGCAGCACGACT
TCTTCAAGTCCGCCATGCCCGAAGGCTACGTCCAGGAGCGCACCATCTTCTTCAAG
GACGACGGCAACTACAAGACCCGCGCCGAGGTGAAGTTCGAGGGCGACACCCTG
GTGAACCGCATCGAGCTGAAGGGCATCGACTTCAAGGAGGACGGCAACATCCTGG
GGCACAAGCTGGAGTACAACTACAACAGCCACAACGTCTATATCATGGCCGACAA
GCAGAAGAACGGCATCAAGGTGAACTTCAAGATCCGCCACAACATCGAGGACGG
CAGCGTGCAGCTCGCCGACCACTACCAGCAGAACACCCCCATCGGCGACGGCCCC
GTGCTGCTGCCCGACAACCACTACCTGAGCACCCAGTCCGCCCTGAGCAAAGACC
CCAACGAGAAGCGCGATCACATGGTCCTGCTGGAGTTCGTGACCGCCGCCGGGAT
CTAACTCGAGCAAAGCCCGCCGAAAGGCGGGCTTTTCTGTGTCGAC

Figure S1. Top: schematic of the plasmid construction P70a-deGFP. **Bottom:** P70a-deGFP coding sequences. The promoter -35 and -10 sequences are bold underlined. The transcription start is the only lowercase letter 'a' just before the NheI site. The UTR (including the RBS) is in italic. The gene *degfp* is in green. The transcription terminator T500 is in red. Some restriction sites are underlined (SphI, NheI, XhoI, SalI)). This plasmid is available at AddGene under the name pBEST-OR2-OR1-UTR1-deGFP-T500 (#40019).

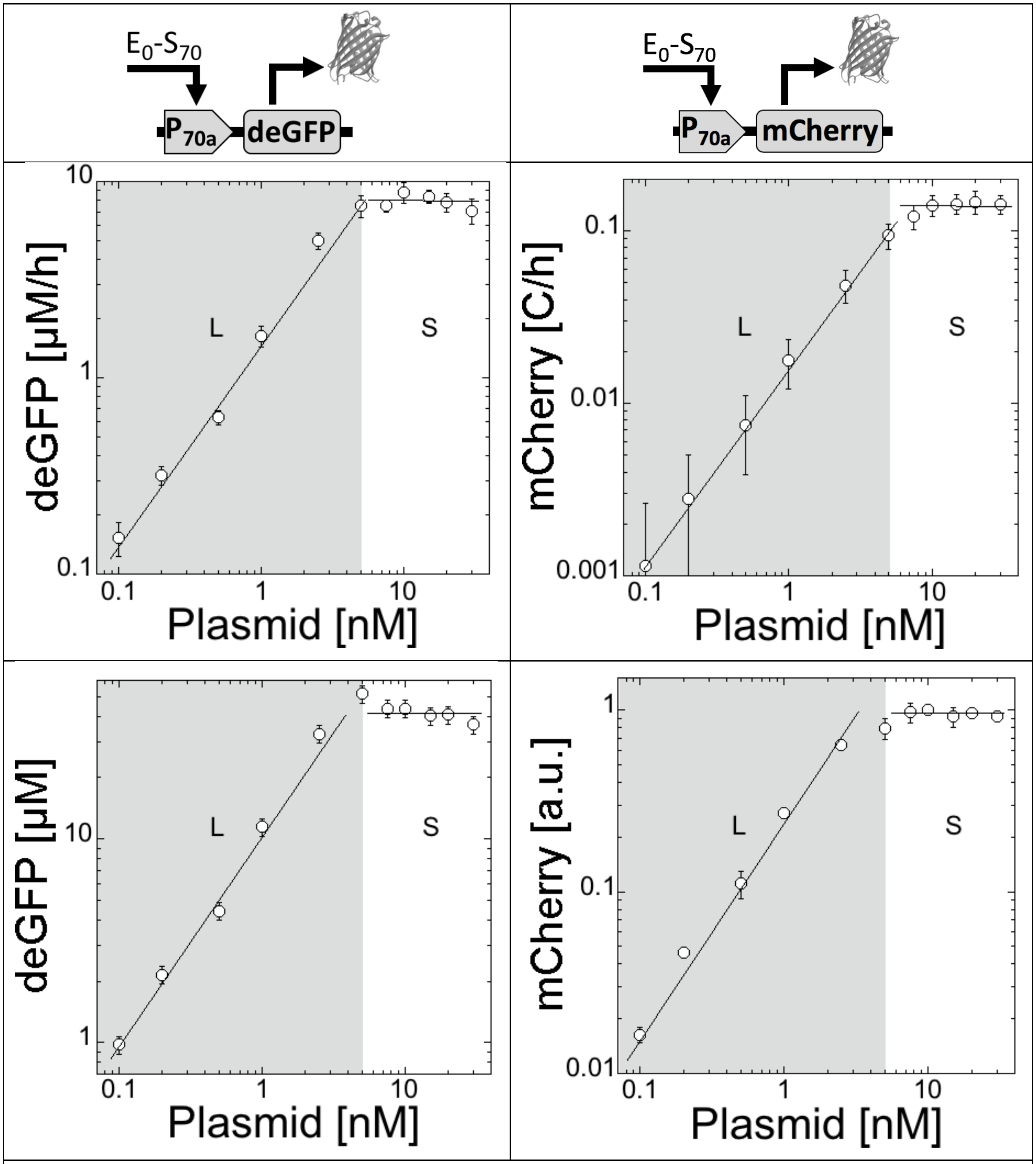

Figure S2. Left column: schematic of the P70a-deGFP plasmid. Rates of deGFP synthesis as a function of the plasmid concentration (as shown in Fig. 1 and 2). L: linear regime, S: saturated regime. **Bottom left:** deGFP synthesis yield after 10 h of incubation as a function of the plasmid concentration. **Right column:** the same circuit and experiments for P70a-mCherry. The sequence of P70a-mCherry is identical to the one in Figure S1 except that mCherry replaces deGFP. Note that for mCherry, units on the Y axes are arbitrary because we had no calibration for this reporter protein.

Visualizing the mRNA steady state was only possible for the first hour because neither the dye used for the fluorescent broccoli RNA aptamer nor malachite green aptamer were stable in the cell-free reaction (data not shown). The figure below shows that mRNA reaches a plateau after 1 h on average. Above 20 µM, the dyes were toxic for the reaction.

GCATGCTGAGCTAACACCGTGCGTG**TTGACA**ATTTTACCTCTGGCGGT**GATAAT**G
GTTGCaGCTAGC*AATAATTTTGTTTAACTTTAAGAAGGAGATATACC*ATGGAGCTTTTCA
CTGCGTTGTTCCCATCCTGGTCGAGCTGGACGGCGACGTAAACGGCCACAAGTTC
AGCGTGTCCGGCGAGGGCGAGGGCGATGCCACCTACGGCAAGCTGACCCTGAAGT
TCATCTGCACCACCGGCAAGCTGCCCGTGCCCTGGCCCACCCTCGTGACCACCCTG
ACCTACGGCGTGCAGTGCTTCAGCCGCTACCCCGACCACATGAAGCAGCACGACT
TCTTCAAGTCCGCCATGCCCGAAGGCTACGTCCAGGAGCGCACCATCTTCTTCAAG
GACGACGGCAACTACAAGACCCGCGCCGAGGTGAAGTTCGAGGGCGACACCCTG
GTGAACCGCATCGAGCTGAAGGGCATCGACTTCAAGGAGGACGGCAACATCCTGG
GGCACAAGCTGGAGTACAACTACAACAGCCACAACGTCTATATCATGGCCGACAA
GCAGAAGAACGGCATCAAGGTGAACTTCAAGATCCGCCACAACATCGAGGACGG
CAGCGTGCAGCTCGCCGACCACTACCAGCAGAACACCCCCATCGGCGACGGCCCC
GTGCTGCTGCCCGACAACCACTACCTGAGCACCCAGTCCGCCCTGAGCAAAGACC
CCAACGAGAAGCGCGATCACATGGTCCTGCTGGAGTTCGTGACCGCCGCCGGGAT
CTAACTCGAGGGGATCCCGACTGGCGAGAGCCAGGTAACGAATGGATCCCAAAG
CCCGCCGAAAGGCGGGCTTTTCTGTGTCGAC

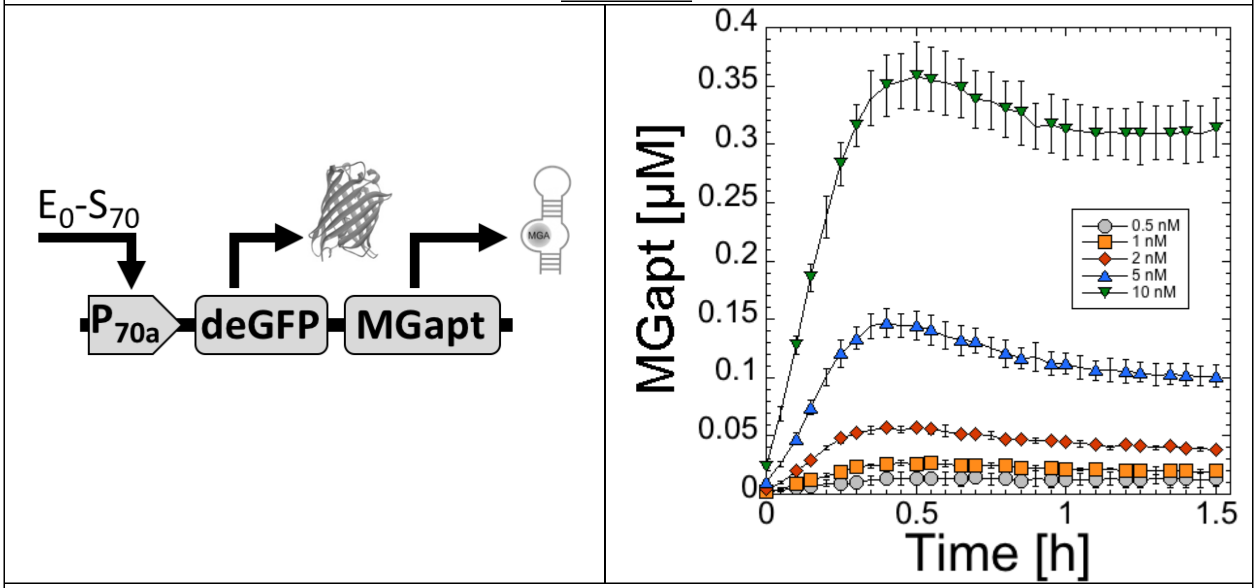

Figure S3. Top: the P70a-deGFP-MGapt coding sequences. The promoter -35 and -10 sequences are bold underlined. The start transcription is the only lowercase letter 'a' just before the NheI site. The UTR (including the RBS) is in italic. The gene *degfp* is in green. The Malachite green aptamer (MGapt) is in purple. The transcription terminator T500 is in red. Some restriction sites are underlined (SphI, NheI, SalI). **Bottom left:** schematic of the construction. **Bottom right:** fluorescence signal of the MGapt for plasmid concentrations ranging from 0.5 nM to 10 nM.

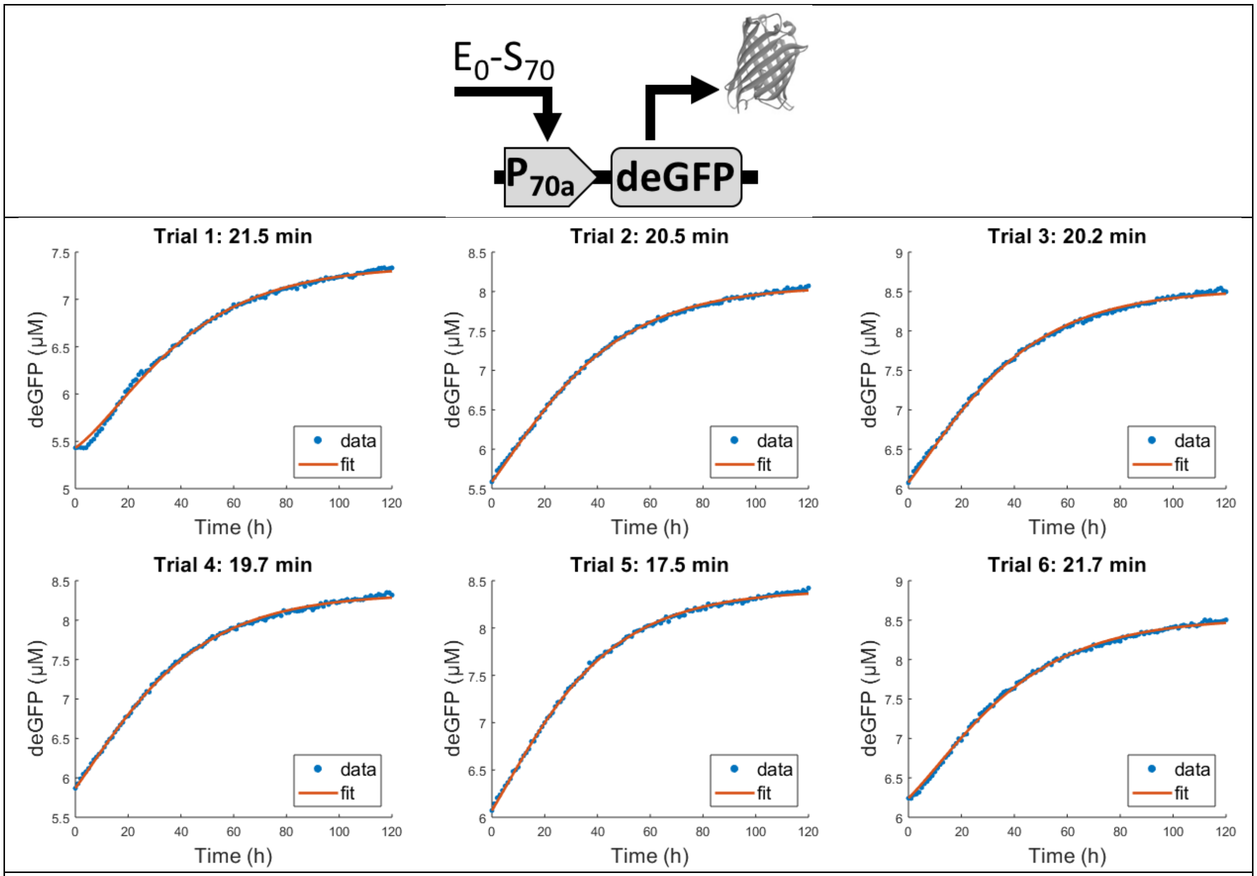

Figure S4. Kinetics of deGFP synthesis after inhibiting transcription to estimate the deGFP mRNA mean lifetime.

We determined the mRNA mean lifetime using an assay described previously[1]. 60 μM Rifampicin completely arrests transcription. We incubated TXTL reactions containing 2 nM P70-deGFP in a 96 well v-bottom plate for two hours at 29°C. After two hours, the reactions are producing deGFP at a constant rate. Then, to one of the reactions, we added 60 μM Rifampicin to immediately stop transcription, and began measuring the fluorescence of deGFP, and fit the data in Matlab using the following set of equations:

$$\frac{d[m]}{dt} = -b[m]$$

$$\frac{d[P_d]}{dt} = a[m] - \kappa[P_d]$$

$$\frac{d[P_f]}{dt} = \kappa[P_d]$$

We arrive at these equations by setting the transcription rate to zero. This last equation has the solution:

$$[P_f](t) = [P_f]_0 + [P_d]_0(1 - e^{-\kappa t}) + \frac{am_0}{b(b-\kappa)}[b(1 - e^{-\kappa t}) + \kappa(e^{-bt} - 1)]$$

where $[P_f](t)$ is the mature, fluorescent deGFP protein as a function of time; $[P_f]_0$ is the initial mature, fluorescent deGFP protein at transcription arrest; $[P_d]_0$ is the initial dark, non-fluorescent deGFP protein at transcription arrest; κ is the deGFP protein maturation rate (23 min); a is a protein synthesis or translation constant; m_0 is the initial mRNA concentration at transcription arrest; and b is the mRNA inactivation rate. The product am_0 is the initial slope of the deGFP kinetics at transcription arrest. The inverse of the mRNA inactivation rate is the mRNA lifetime, which was found to be 20.2 ± 1.5 min from 6 technical replicates.

The transcription speed (C_m) in cell-free reactions was estimated by expressing the MG aptamer on different sized transcripts. We cloned the MG aptamer on the 3' end of transcripts with lengths of 326, 826, 1326, and 1826 nucleotides, under the P70a promoter (the MG aptamer itself is an additional 39 nucleotides). To determine the speed of transcription, we measured the time it takes for the first RNAs of different lengths to be fully synthesized (Fig. S5). This was done by immediately measuring the fluorescence after adding malachite green and the plasmid to a cell-free reaction. Exactly one minute passed between the addition of the plasmid and the first fluorescence measurement. After about four minutes of measuring, we see that the fluorescence kinetics appear roughly linear.

We took a linear fit of the points between 4-6 minutes, and extrapolated the fit to 0 fluorescence to get an approximation for the time of the first bursts of RNA, adding on the one minute before measurements started. We ran five trials for each transcript length. Taking the difference in times of the initial RNA burst for each of the different transcript lengths, we can fit them on the same plot and determine the average RNA polymerase transcription speed, which we found to be 9.8 ± 1.8 nucleotides per second (Fig. S5).

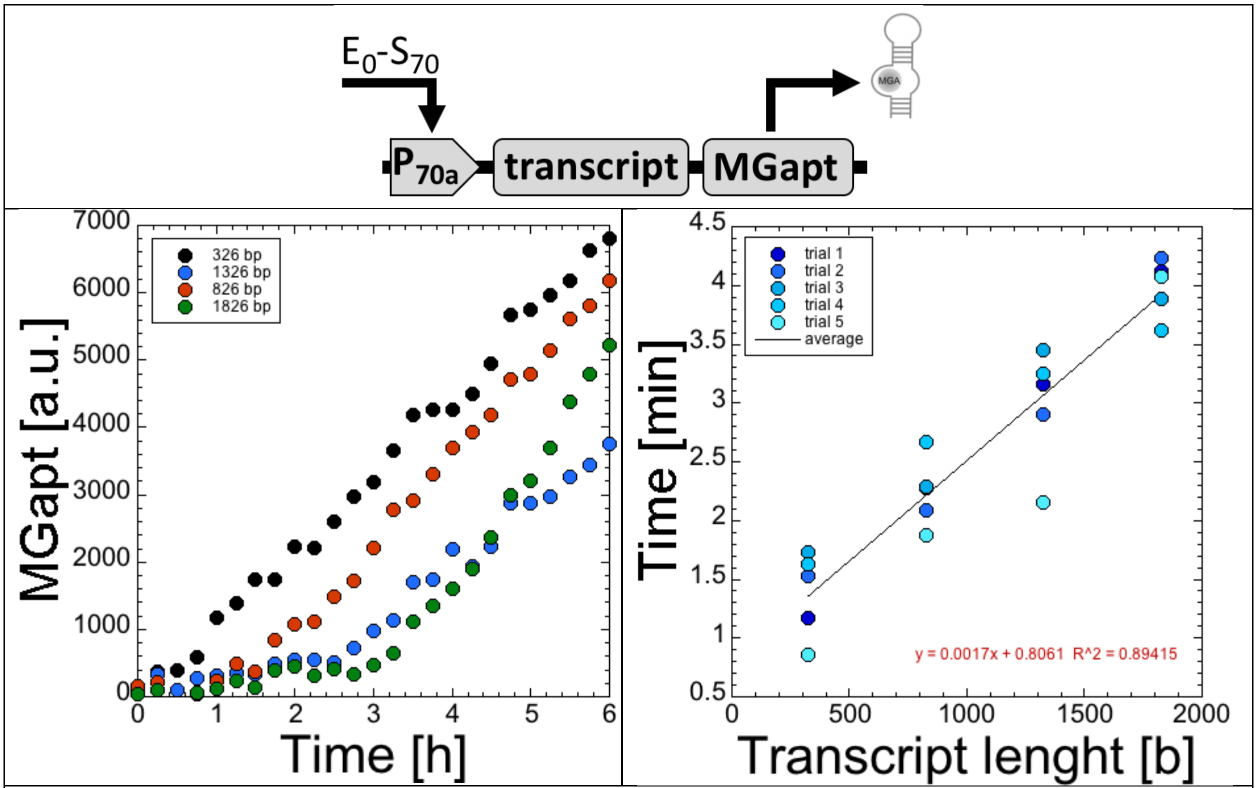

Figure S5. Top: Schematic of the construction. **Bottom left:** example of MG aptamer kinetics for the four different transcript lengths. **Bottom right:** 5 trials were performed and linear fit.

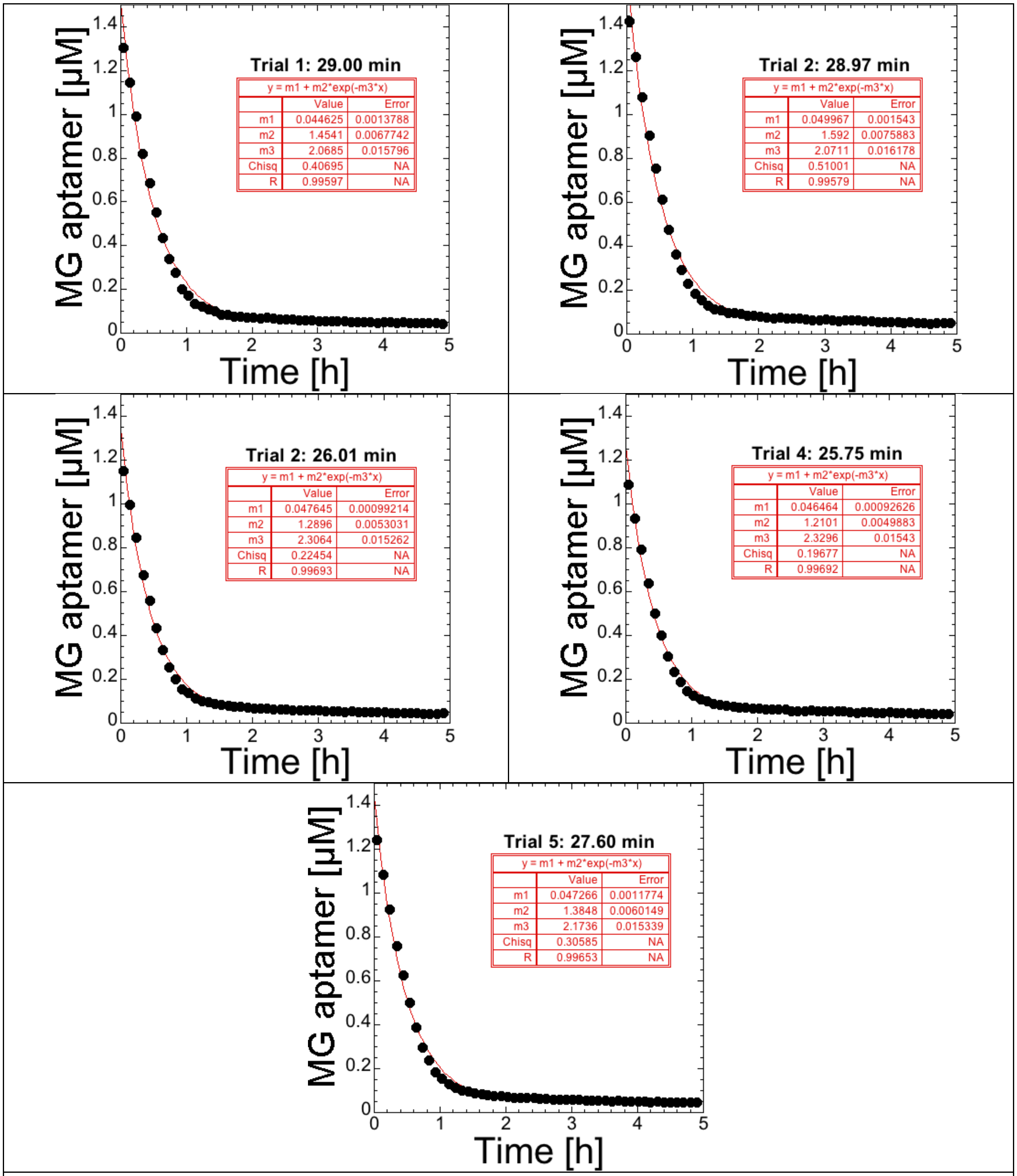

Figure S6. Degradation of the MG aptamer in TXTL reactions. The MG aptamer was synthesized using an *in vitro* transcription kit and quantified using a nanodrop. The pure MG aptamer was added to TXTL reactions at concentrations of 1.5 μM (5 trials) and the rate of degradation was measured and fit by an exponential decay: $d[m]/dt = -k_{dm} * [m]$, where $1/k_{dm} = 27.47 \pm 1.56$ minutes (average of 5 trials).

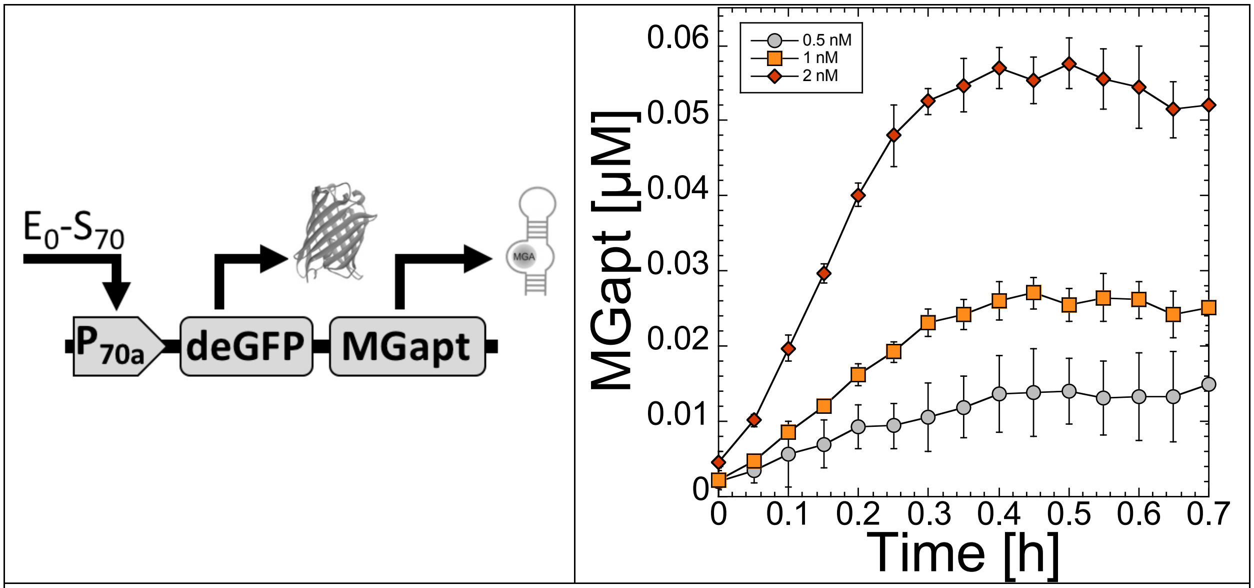

Figure S7. Left: schematic of P70a-deGFP-MGapt plasmid. **Right:** kinetics of MGapt accumulation reaching steady state after about 1 h for three plasmid concentrations: 0.5, 1 and 2 nM. The MGapt concentration at steady state scale linearly with the plasmid concentration. For 1 nM and 2 nM plasmid we get around 25 nM and 50 nM MGapt at steady state, respectively. Taking this numerical value in Eq. 27 and a lifetime for MGapt of 27 min, we get that $k_{cat,m} \approx k_{TX} = 1.5\ 10^{-2}\ s^{-1}$.

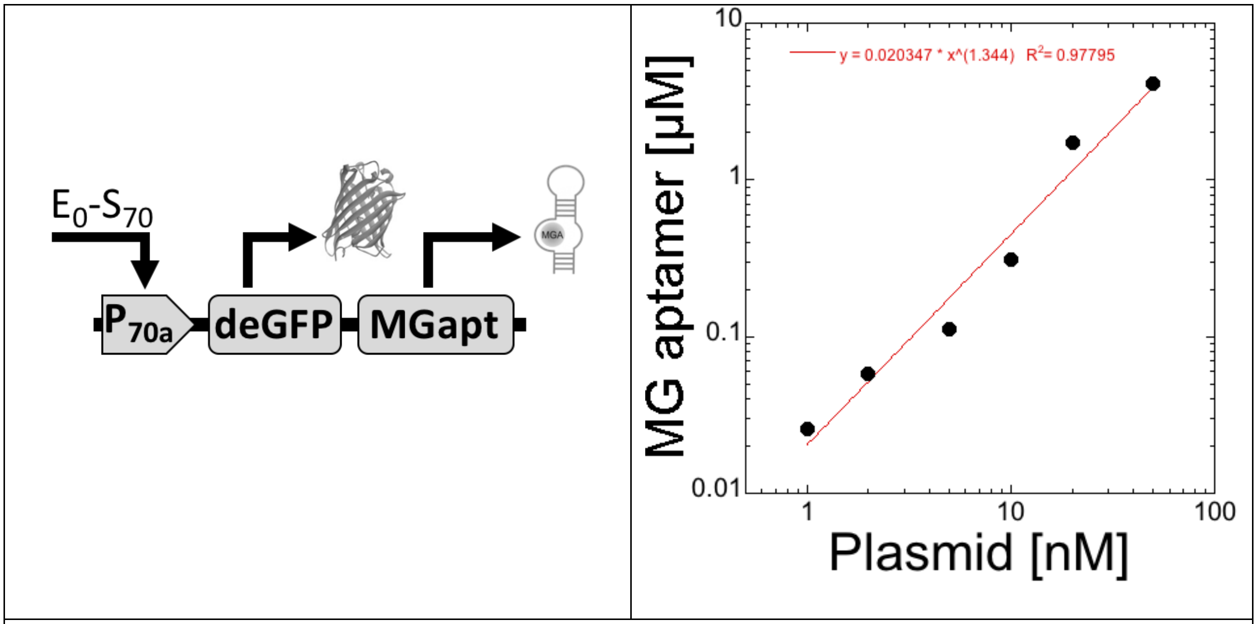

Figure S8. Left: schematic of the plasmid P70a-deGFP-MGapt. **Right:** mRNA synthesis never saturates (plasmid concentrations: 1, 2, 5, 10, 20, 50 nM). This plot shows the concentration of MG aptamer after 1 h of incubation.

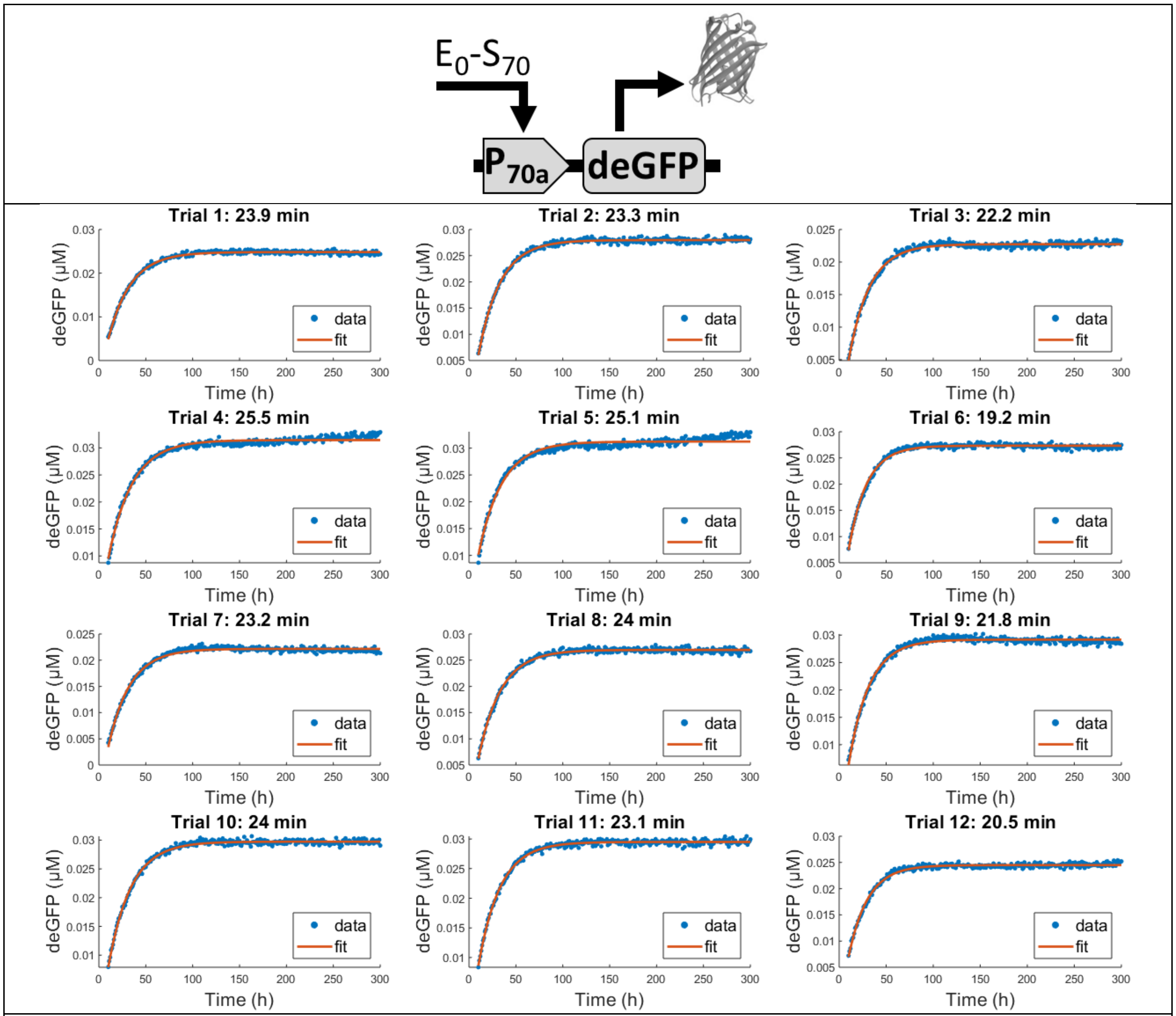

Figure S9. Top: schematic of the circuit. **Bottom:** Kinetics of deGFP synthesis (P70a-deGFP, 5 nM) after addition of RNAse A to measure the maturation time of the reporter protein.

We determined the maturation time of deGFP using an assay described previously[1]. RNAse A completely and instantaneously arrests translation[2]. There is also no GFP produced in the first 150 seconds after adding DNA. We incubated a 2 µl TXTL reaction (assembled with the Labcyte Echo 550) in a 96 well v-bottom plate without DNA or RNAse for 30 minutes at 29°C to bring the reactions at the right temperature. Then, we added 5 nM P70a-deGFP and incubated for 3 minutes before adding RNAse A to instantaneously stop translation. We immediately started measuring fluorescence of deGFP, and fit the data with Matlab using the equation (P_f: fluorescent protein, P_d: dark protein):

$$\frac{d[P_f]}{dt} = \kappa[P_d]$$

We arrive at this equation because we can set the translation rate to zero. This equation has the solution:

$$[P_f](t) = [P_f]_0 + [P_d]_0(1 - e^{-\kappa t})$$

where $[P_f](t)$ is the mature, fluorescent deGFP protein as a function of time; $[P_f]_0$ is the initial mature, fluorescent deGFP protein at translation arrest, which in this case is zero; $[P_d]_0$ is the initial dark, non-fluorescent deGFP protein at translation arrest (fit by Matlab); and κ is the deGFP protein maturation rate. The inverse of this rate is the maturation time, which was found to be 23 ± 1.8 min from 12 technical replicates.

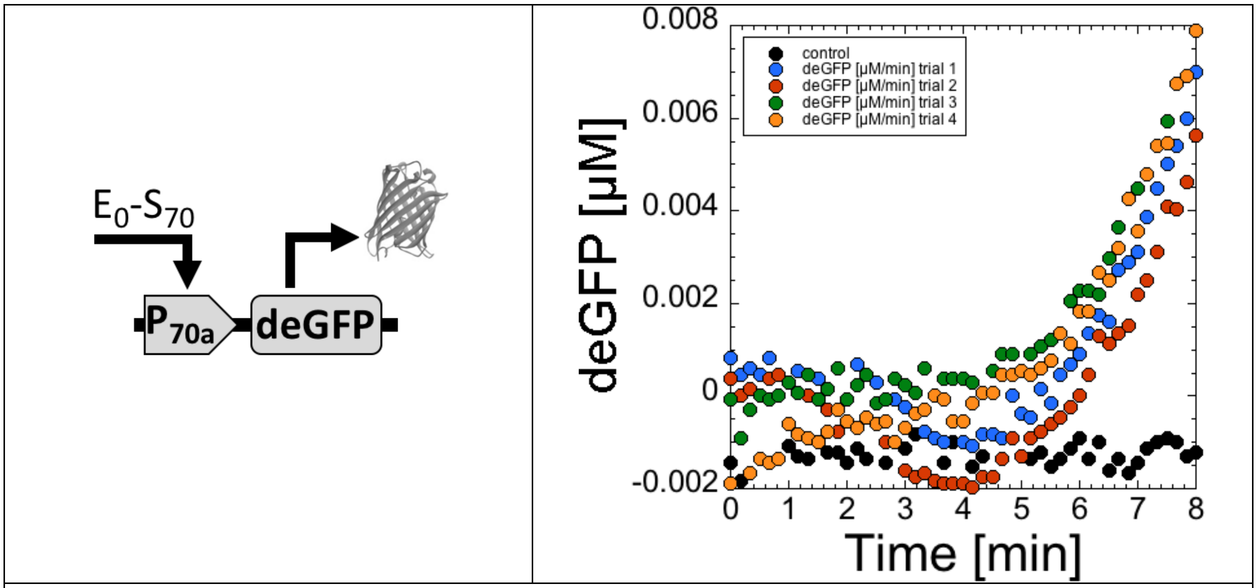

Figure S10. deGFP synthesis was measured in the first few minutes of incubation to estimate the TL speed (C_p). The lower bound on translation speed was estimated in cell-free reactions by measuring the time until the first fluorescence signal when expressing deGFP from the P70a promoter (5 nM). The first fluorescent deGFP proteins were measured after about 4.5 minutes, and the coding sequence is 675 nt long; therefore, the minimum translation speed is 2.5 nt/s, or just under 1 amino acid per second. This measurement provides a lowest limit to C_p because it does not take into consideration transcription, the protein folding (even for the first reporter proteins) and the limit of detection. The translation speed has been estimated to be around 2 amino acids per second in other studies[3].

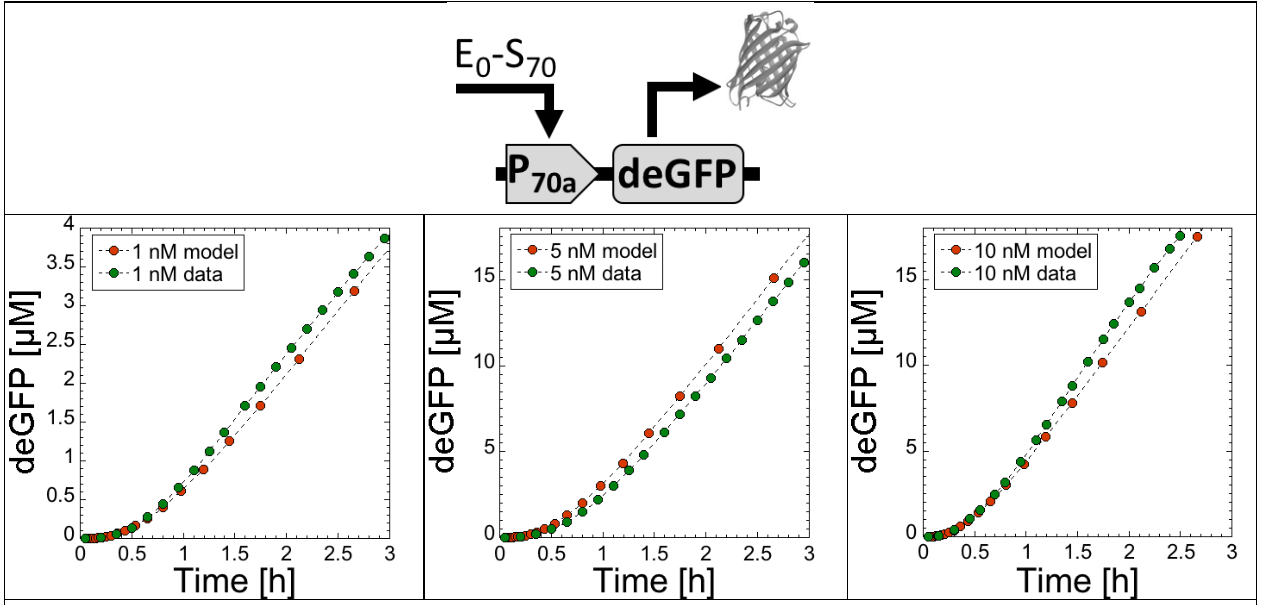

Figure S11. Kinetics of deGFP synthesis for the plasmid P70a-deGFP at three concentrations: one below, one at, and one above saturation (1 nM, 5 nM, 10 nM), shown for the first 3 hours.

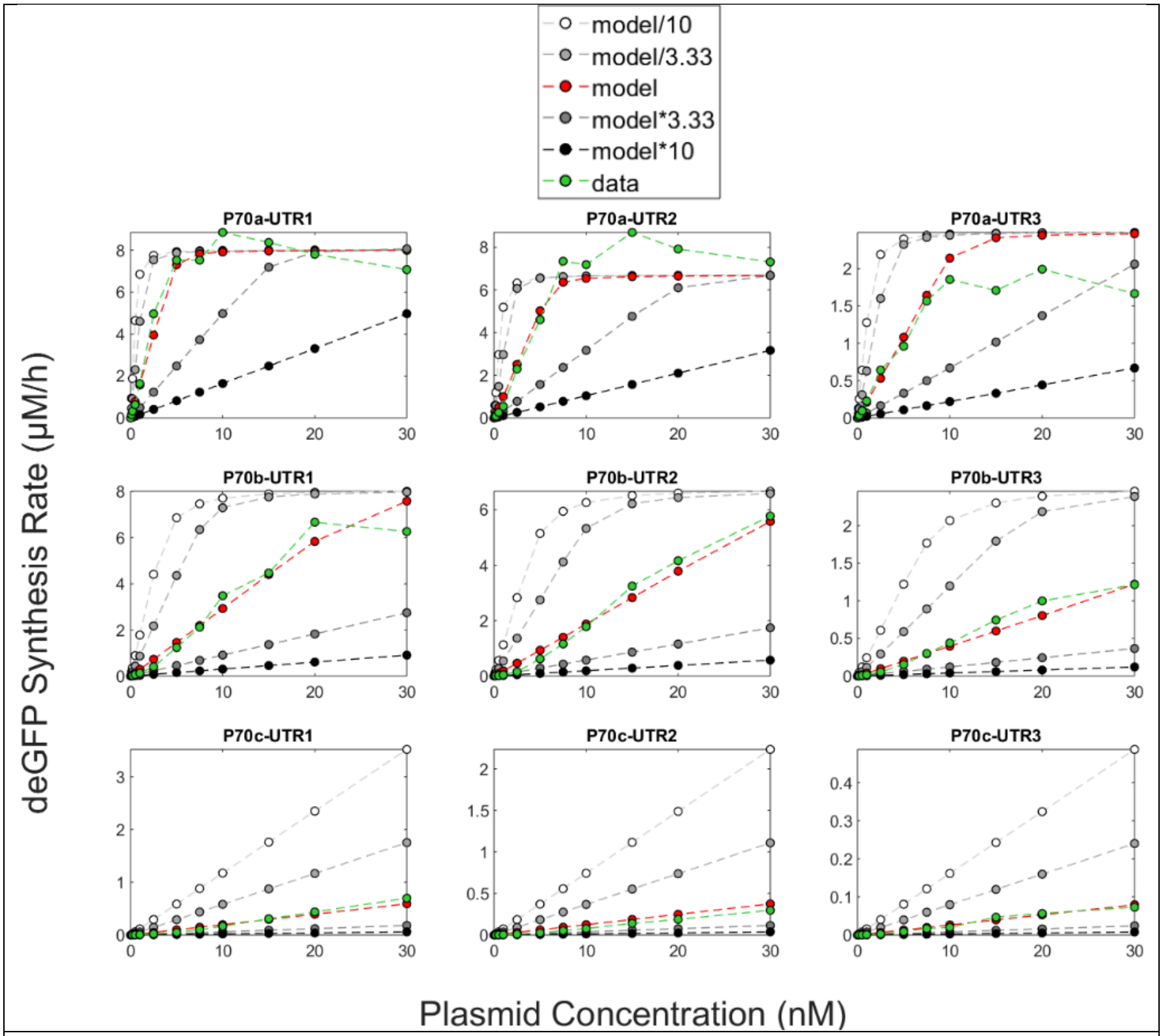

Figure S12. Model Sensitivity to changing the deGFP mRNA degradation rate $k_{deg,m}$ for combinations of three promoters and UTRs for the four different rates (/10, /3.33, *3.33, *10) in addition to the best fit ($k_{deg,m}$ = 1/20.2 min for the best fit, $k_{deg,m} = k_{d,m} / K_{M,m}$). A large sensitivity is observed for all the cases.

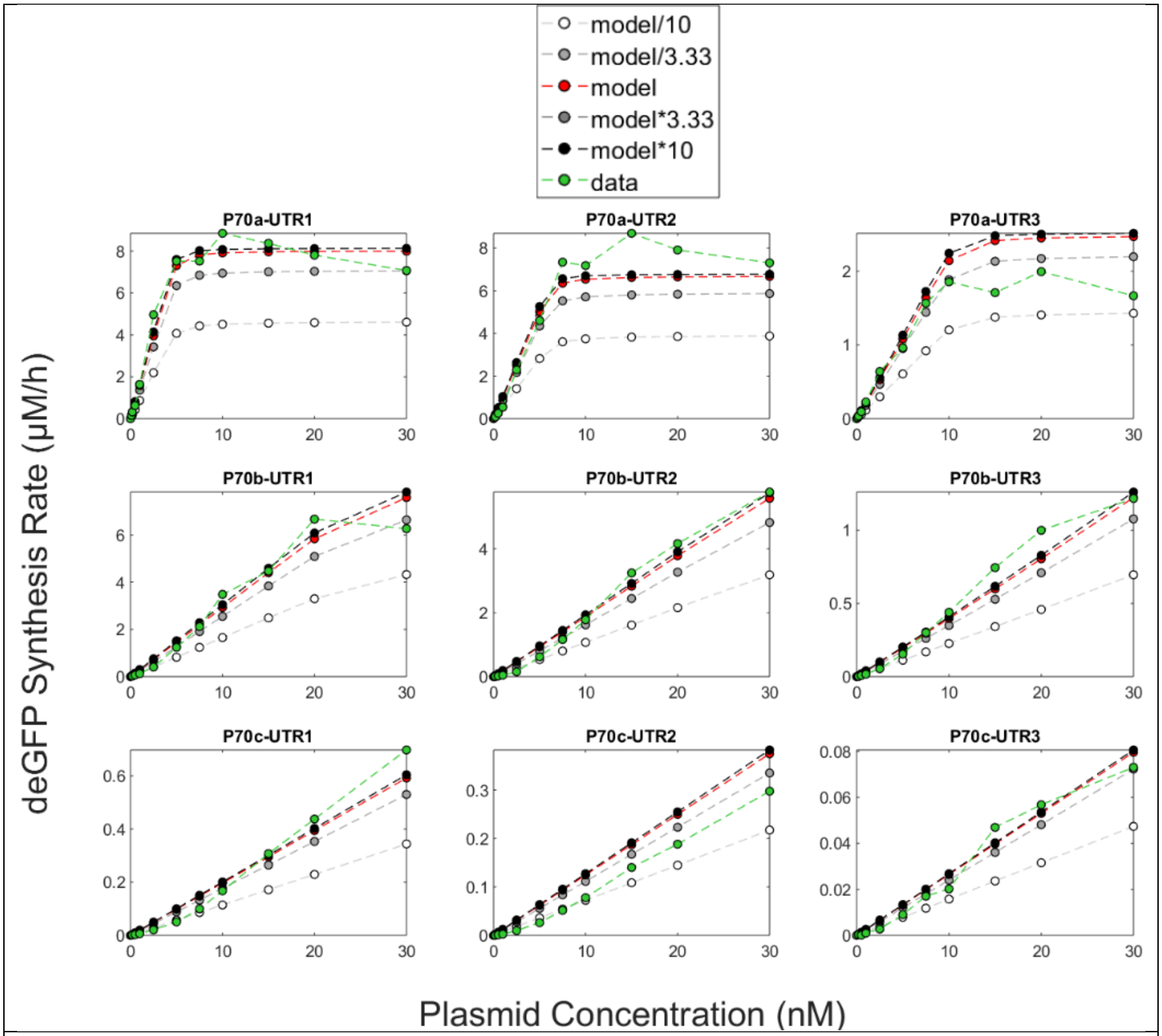

Figure S13. Model Sensitivity to changing the deGFP maturation rate k_{mat} for combinations of three promoters and UTRs for the four different maturation times (/10, /3.33, *3.33, *10) in addition to the best fit (k_{mat} = 7.25 10^{-4} s^{-1} for the best fit). Some sensitivity is observed, especially when the maturation time is 10 times smaller.

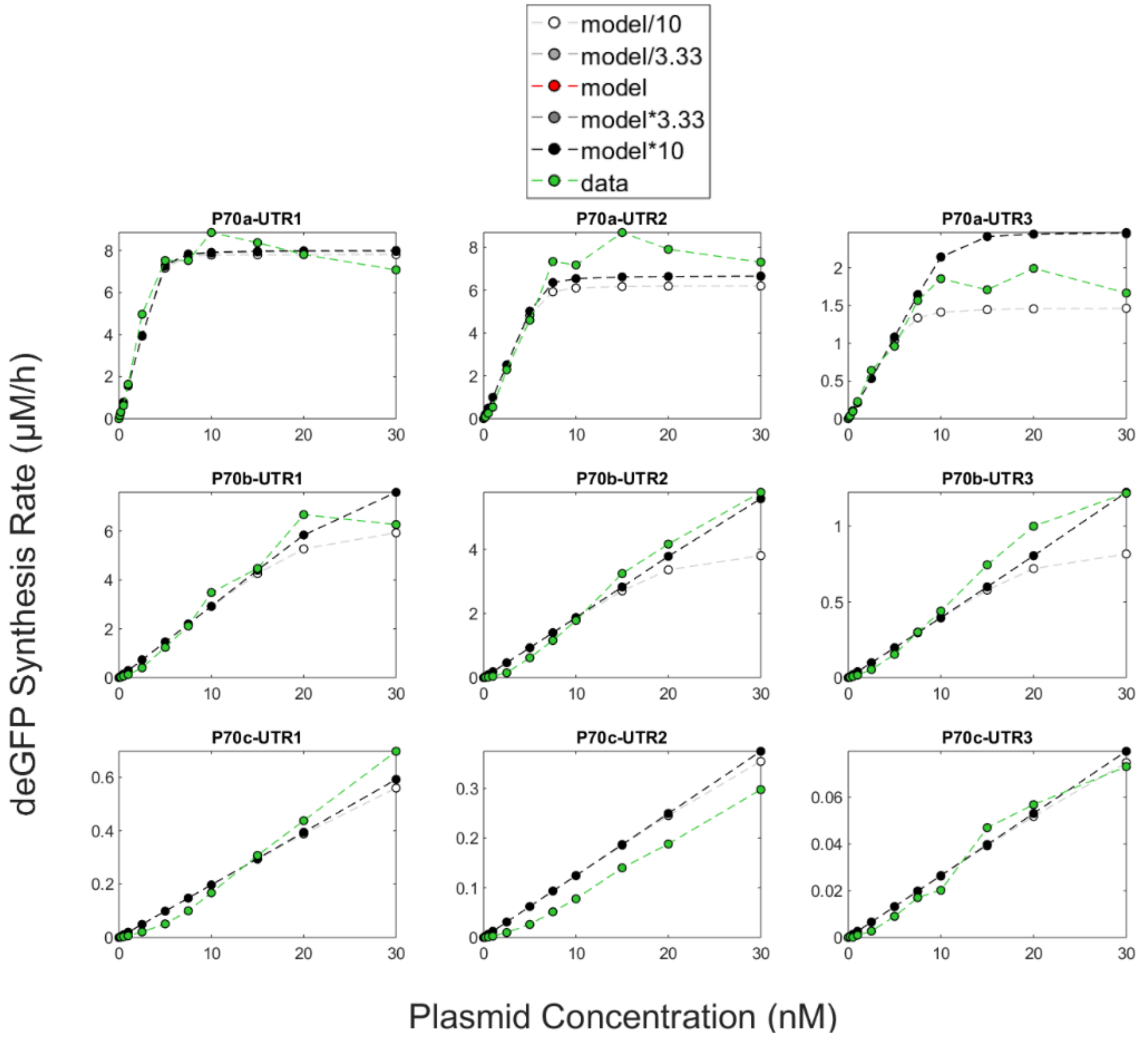

Figure S14. Model Sensitivity to changing the concentration of core RNA polymerase E_{tot} for combinations of three promoters and UTRs for the four different concentrations of total RNA polymerase (/10, /3.33, *3.33, *10) in addition to the best fit (E_{tot} = 400 nM for the best fit). Some sensitivity is observed for P70a and P70b, with UTR2 and UTR3, when the concentration of core RNA polymerase E_{tot} is much smaller (/10). Note that for the nine plots, the cases *10, *3.33, model, /3.33 are all superimposed.

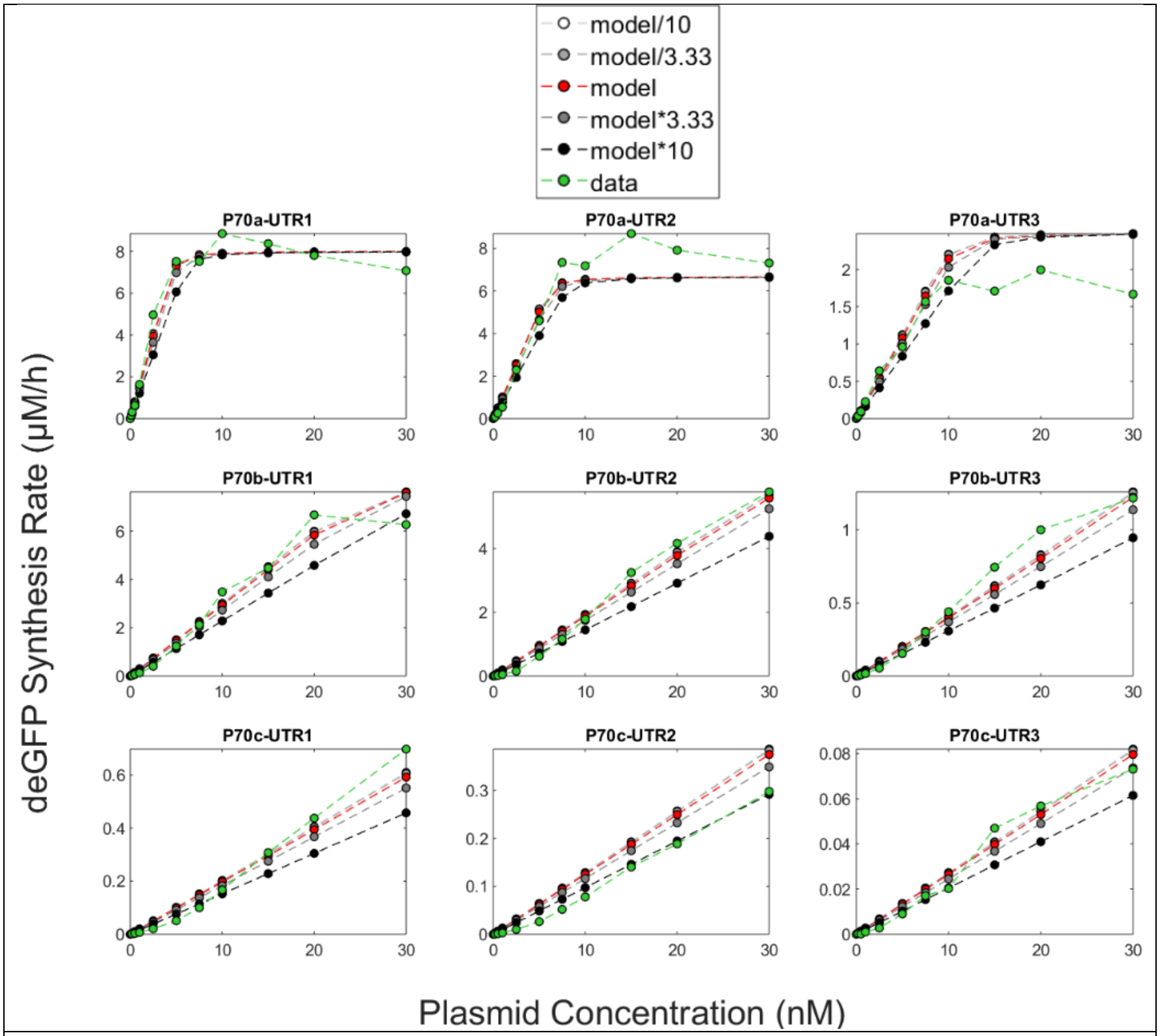

Figure S15. Model Sensitivity to changing the Michaelis-Menten constant for transcription $K_{M,70}$ for combinations of three promoters and UTRs for the four different values of the constant (/10, /3.33, *3.33, *10) in addition to the best fit ($K_{M,70}$ = 1 nM for the best fit). No sensitivity is observed.

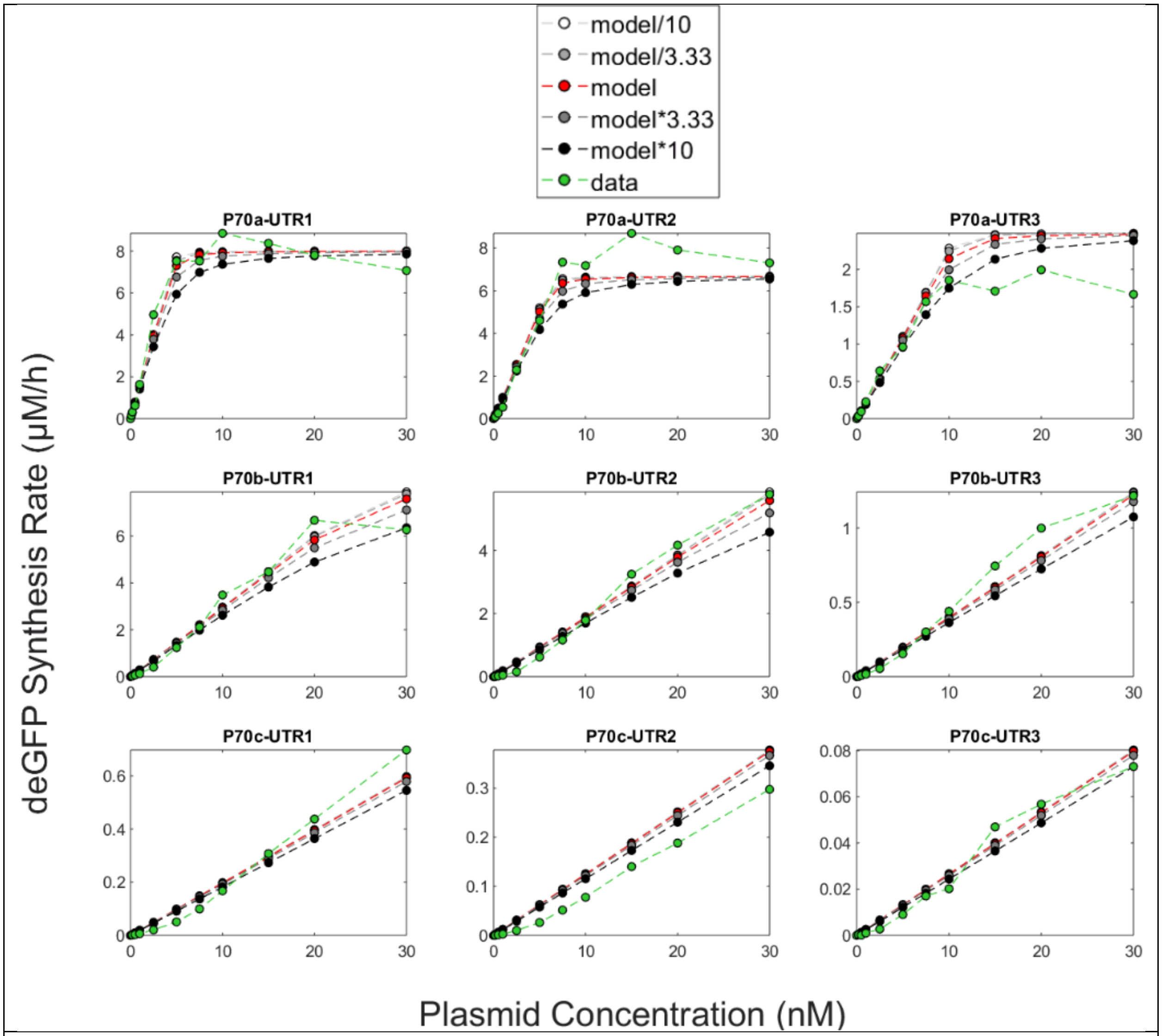

Figure S16. Model Sensitivity to changing the Michaelis-Menten constant for translation $K_{M,R}$ for combinations of three promoters and UTRs for the four different values of the constant (/10, /3.33, *3.33, *10) in addition to the best fit ($K_{M,R}$ = 10 nM for the best fit). No sensitivity is observed.

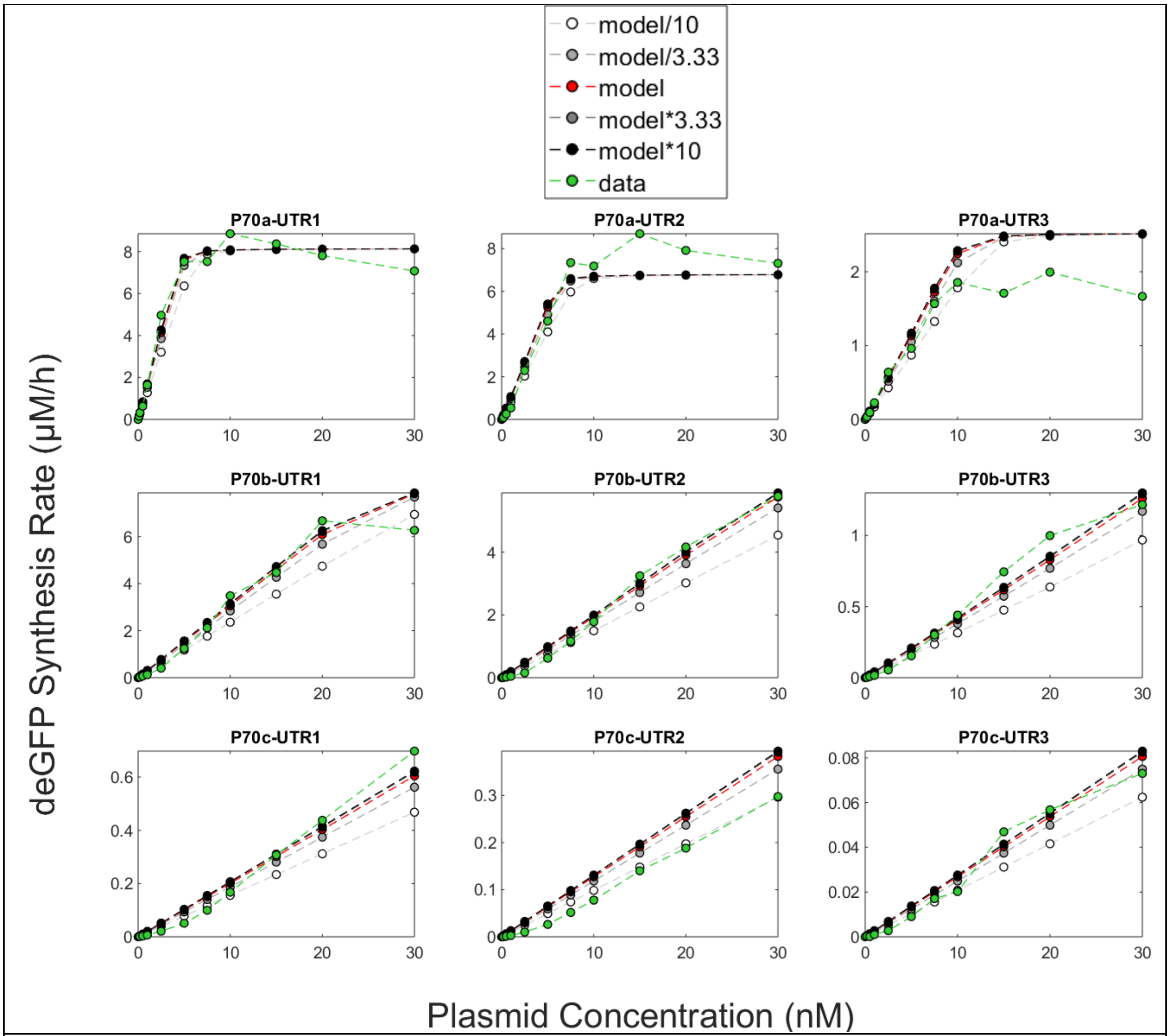

Figure S17. Model Sensitivity to changing the concentration of sigma factor 70 for combinations of three promoters and UTRs for the four different concentrations of total sigma factor 70 (/10, /3.33, *3.33, *10) in addition to the best fit ($S70_t$ = 30 nM for the best fit). Very little sensitivity is observed for any of the promoter and UTR combinations.

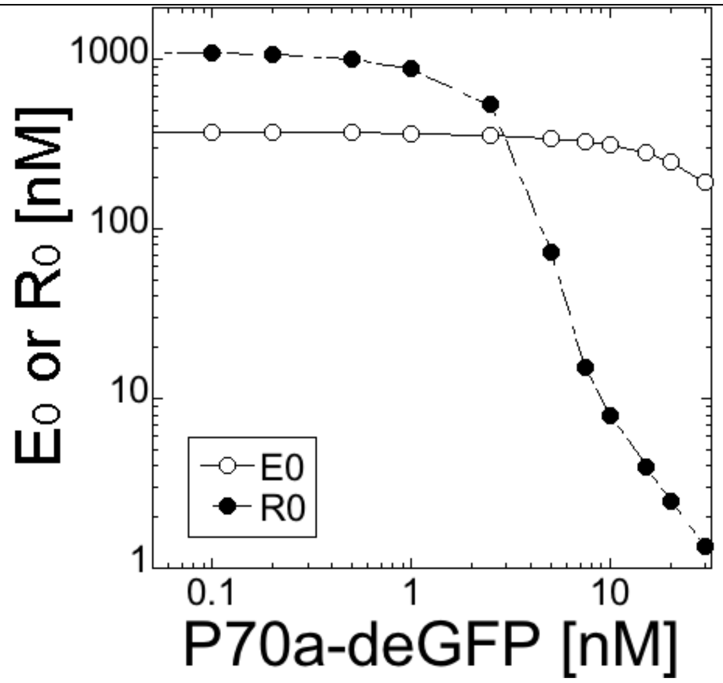

Figure S18. Model predictions of the concentrations of free core RNA polymerase E_0 and free ribosomes R_0 at steady state with respect to the concentration of plasmid P70a-deGFP in a cell-free reaction. E_{tot} = 400 nM and R_{tot} = 1100 nM. Saturation due to transcription occurs at a plasmid concentration ten times larger than for translation.

DNA sequences of the regulatory parts used in this work (promoters and UTRs), from SphI restriction site to the ATP of the *degfp* gene. The promoter -35 and -10 sequences are bold underlined. The start transcription is the only lowercase letter ‘a’ just before the NheI site. The UTR (including the RBS) is in italic. The ATG of the *degfp* gene is in green. These regulatory sequences were cloned in P70a-dGFP available at Addgene (plasmid #40019) under the name pBEST-OR2-OR1-Pr-UTR1-deGFP-T500 (see Fig. S1).

P70a-UTR1:
GCATGCTGAGCTAACACCGTGCGTG**TTGACA**ATTTTACCTCTGGCGGT**GATAAT**GGTTGCaG
CTAGC*AATAATTTTGTTTAACTTTAAGAAGGAGATATACC*ATG

P70b-UTR1:
GCATGCTGAGCTAACACCGTGCGTG**TTTACA**ATTTTACCTCTGGCGGT**GATAAT**GGTTGCaG
CTAGC*AATAATTTTGTTTAACTTTAAGAAGGAGATATACC*ATG

P70c-UTR1:
GCATGCTGAGCTAACACCGTGCGTG**TTGACA**ATTTTACCTCTGGCGGT**GATAAA**GGTTGCaG
CTAGC*AATAATTTTGTTTAACTTTAAGAAGGAGATATACC*ATG

P70a-UTR2:
GCATGCTGAGCTAACACCGTGCGTG**TTGACA**ATTTTACCTCTGGCGGT**GATAAT**GGTTGCaG
CTAGC*AATAATTTTGTTTAACTTTAAGAAGGATATATACC*ATG

P70b-UTR2:
GCATGCTGAGCTAACACCGTGCGTG**TTTACA**ATTTTACCTCTGGCGGT**GATAAT**GGTTGCaG
CTAGC*AATAATTTTGTTTAACTTTAAGAAGGATATATACC*ATG

P70c-UTR2:
GCATGCTGAGCTAACACCGTGCGTG**TTGACA**ATTTTACCTCTGGCGGT**GATAAA**GGTTGCaG
CTAGC*AATAATTTTGTTTAACTTTAAGAAGGATATATACC*ATG

P70a-UTR3:
GCATGCTGAGCTAACACCGTGCGTG**TTGACA**ATTTTACCTCTGGCGGT**GATAAT**GGTTGCaG
CTAGC*AATAATTTTGTTTAACTTTAAGAAGGGGGTATACC*ATG

P70b-UTR3:
GCATGCTGAGCTAACACCGTGCGTG**TTTACA**ATTTTACCTCTGGCGGT**GATAAT**GGTTGCaG
CTAGC*AATAATTTTGTTTAACTTTAAGAAGGGGGTATACC*ATG

P70c-UTR3:
GCATGCTGAGCTAACACCGTGCGTG**TTGACA**ATTTTACCTCTGGCGGT**GATAAA**GGTTGCaG
CTAGC*AATAATTTTGTTTAACTTTAAGAAGGGGGTATACC*ATG

PrpoH-UTR1:

GCATGCGGTACAACATTTACGCCAC**TTTACG**CCTGAATAATAAAAGCGTGT**TATACT**CTTTC
CCGCTAGC*AATAATTTTGTTTAACTTTAAGAAGGAGATATACC*ATG

PrecA-UTR1:
GCATGCAACAATTTCTACAAAACAC**TTGATA**CTGTATGAGCATACAG**TATAAT**TGCTTGCTA
GC*AATAATTTTGTTTAACTTTAAGAAGGAGATATACC*ATG

PrrsB-UTR1:
GCATGCAATTATTTTAAATTTCCTC**TTGTCA**GGCCGGAATAACTCCC**TATAAT**GCGCCACCG
CTAGC*AATAATTTTGTTTAACTTTAAGAAGGAGATATACC*ATG

PlacI-UTR1:
GCATGCCGTTGACACCATCGAATGGCGCAAAACCTTTCGCGGTATGG**CATGAT**AGCGCCCG
GGCTAGC*AATAATTTTGTTTAACTTTAAGAAGGAGATATACC*ATG

P70a-UTRacpP:
GCATGCTGAGCTAACACCGTGCGTG**TTGACA**ATTTTACCTCTGGCGGT**GATAAT**GGTTGCaG
CTAGC*AACCATCGCGAAAGCGAGTTTTGATAGGAAATTTAAGAGT*ATG

P70a-UTRrpsA:
GCATGCTGAGCTAACACCGTGCGTG**TTGACA**ATTTTACCTCTGGCGGT**GATAAT**GGTTGCaG
CTAGC*CGCGCCAGAAATTGGCTCTCGCATAAGCGACCGAATTTGCAGTACCCCCGTTGCAATGGA*
ATGACAGCGGGTATGTTAAACAACCCCATCCGGCATGGAGCCAGGTGGACGTTAAATATAAACCTG
*AAGATTAAAC*ATG

P70a-UTRrpoH:
GCATGCTGAGCTAACACCGTGCGTG**TTGACA**ATTTTACCTCTGGCGGT**GATAAT**GGTTGCaG
CTAGC*TACCACTGAAGCGCCAGAAGATATCGATTGAGAGGATTTGA*ATG

P70a-UTRlacI:
GCATGCTGAGCTAACACCGTGCGTG**TTGACA**ATTTTACCTCTGGCGGT**GATAAT**GGTTGCaG
CTAGC*AAGAGAGTCAATTCAGGGTGGTGAATCC*ATG

Example of Matlab code:

```
function dy=eqs(t,y)
dy=zeros(2,1);
P70=0;  % concentration of promoter P70 [nM]
P71=.1;
P72=.2;
P73=.5;
P74=1;
P75=2.5;
P76=5;
P77=7.5;
P78=10;
P79=15;
P80=20;
P81=30;
global gg;
    if gg==2 || gg==3 || gg==4
        kcm=0.065; % P70a transcription rate [1/s]
    elseif gg== 5 || gg==6 || gg==7
        kcm=0.012; %P70b
    elseif gg==8 || gg==9 || gg==10
        kcm=0.0008; %P70c
    end
Km=1;    % MM constant for mRNA synthesis [nM]
S70t=30;  % total concentration of sigma70 [nM]
kdm=6.6; % rate of mRNA degradation, nM/s
Krna=8000; %  MMs constant for RNA degradation
    if gg==2 || gg==5 || gg==8
        kcp=0.006; % translation rate constant, UTR1 [s-1]
    elseif gg==3 || gg==6 || gg==9
        kcp=0.0038; %UTR2
    elseif gg==4 || gg==7 || gg==10
        kcp=0.0008; %UTR3
    end
Kp=10;   % translation MM constant [nM]
Et=400;    % total concentration of active core E. coli RNAP [nM]
k70=0.26; % dissociation constant sigma 70 and E. coli core RNAP [nM]
Lm=800;   % length of messenger RNA in nt
Cm=10;     % rate of transcription [nt/s]
Cp=2.5;     % rate of translation [nt/s]
Rt=1100;  % concentration of ribosomes [nM]
kf=0.000725; % rate of protein maturation [s-1]

P70a = [P70 P71 P72 P73 P74 P75 P76 P77 P78 P79 P80 P81];

for i=1:length(P70a) %looping for all concentrations of DNA
    % solving the equation to determine the concentration of free RNA
polymerase

e0=@(E0)E0+E0*S70t/(k70+E0)+E0*S70t*P70a(i)/(E0*S70t+Km*(k70+E0))*(1+kcm*Lm/C
m)-Et;
    E0=fzero(e0,[0,Et]);

    % solving the equation to determine the concentration of free ribosomes
R0
```

```
    r0=@(R0)R0+R0*y(3*i-2)/(R0+Kp)*(1+kcp*Lm/Cp)-Rt;
    R0=fzero(r0,[0,Rt]);
    % differential equations
    dy(3*i-2)=kcm*P70a(i)*E0*S70t/(E0*S70t+Km*(E0+k70))-kdm*y(3*i-
2)/(Krna+y(3*i-2));
    dy(3*i-1)=kcp*y(3*i-2)*R0/(Kp+R0)-kf*y(3*i-1);
    dy(3*i)=kf*y(3*i-1);
end
end
```

References

1. Garamella, J., Marshall, R., Rustad, M. & Noireaux, V. The All E. coli TX-TL Toolbox 2.0: A Platform for Cell-Free Synthetic Biology. *ACS Synth. Biol.* **5**, (2016).
2. Shin, J. & Noireaux, V. Study of messenger RNA inactivation and protein degradation in an Escherichia coli cell-free expression system. *J Biol Eng* **4**, 9 (2010).
3. Underwood, K. A., Swartz, J. R. & Puglisi, J. D. Quantitative polysome analysis identifies limitations in bacterial cell-free protein synthesis. *Biotechnol Bioeng* **91**, 425–435 (2005).
